# Underage JUUL Use Patterns: Content Analysis of Reddit Messages

**DOI:** 10.2196/13038

**Published:** 2019-09-09

**Authors:** Yongcheng Zhan, Zhu Zhang, Janet M Okamoto, Daniel D Zeng, Scott J Leischow

**Affiliations:** 1 Department of Management Information Systems University of Arizona, Tucson Tucson, AZ United States; 2 State Key Laboratory of Management and Control for Complex Systems Institute of Automation Chinese Academy of Sciences Beijing China; 3 Shenzhen Artificial Intelligence and Data Science Institute (Longhua) Shenzhen China; 4 Department of Research Mayo Clinic Phoenix, AZ United States; 5 College of Health Solutions Arizona State University Phoenix, AZ United States

**Keywords:** electronic nicotine delivery systems, social media, minors

## Abstract

**Background:**

The popularity of JUUL (an e-cigarette brand) among youth has recently been reported in news media and academic papers, which has raised great public health concerns. Little research has been conducted on the age distribution, geographic distribution, approaches to buying JUUL, and flavor preferences pertaining to underage JUUL users.

**Objective:**

The aim of this study was to analyze social media data related to demographics, methods of access, product characteristics, and use patterns of underage JUUL use.

**Methods:**

We collected publicly available JUUL-related data from Reddit. We extracted and summarized the age, location, and flavor preference of subreddit UnderageJuul users. We also compared common and unique users between subreddit UnderageJuul and subreddit JUUL. The methods of purchasing JUULs were analyzed by manually examining the content of the Reddit threads.

**Results:**

A total of 716 threads and 2935 comments were collected from the subreddit UnderageJuul before it was shut down. Most threads did not mention a specific age, but ages ranged from 13 years to greater than 21 years in those that did. Mango, mint, and cucumber were the most popular among the 7 flavors listed on JUUL’s official website, and 336 subreddit UnderageJuul threads mentioned 7 discreet approaches to circumvent relevant legal regulations to get JUUL products, the most common of which was purchasing JUUL from other Reddit users (n=181). Almost half of the UnderageJuul users (389/844, 46.1%) also participated in discussions on the main JUUL subreddit and sought information across multiple Reddit forums. Most (64/74, 86%) posters were from large metropolitan areas.

**Conclusions:**

The subreddit UnderageJuul functioned as a forum to explore methods of obtaining JUUL and to discuss and recommend specific flavors before it was shut down. About half of those using UnderageJuul also used the more general JUUL subreddit, so a forum still exists where youths can attempt to share information on how to obtain JUUL and other products. Exploration of such social media data in real time for rapid public health surveillance could provide early warning for significant health risks before they become major public health threats.

## Introduction

### Background

JUUL, the most popular e-cigarette brand in the United States, currently holds 71% of the market share, according to data from Wells Fargo in July 2018 [1]. JUUL’s popularity is due to several characteristics. First, JUUL’s appearance is similar to a universal serial bus (USB), which makes it easy to carry and hide as a portable device. Compared with traditional cigarette appearance, USB-like appearance has less of a negative perceived social image in public locations. Second, nicotine delivery via JUUL is effective; it is sufficiently high to warrant concern about development of nicotine addiction [2]. Third, JUUL offers a wide array of flavor options. JUUL’s manufacturer claims “JUUL is for adult smokers seeking a satisfying alternative to cigarettes.” However, a recent survey reported 8% of Americans aged 15 to 24 years used JUUL in the 30 days before the survey [3]. The popularity of JUUL among youth has also recently been reported in news media [4-6]. Substantial and growing evidence shows that e-cigarette use by youth and young adults increases the risk of ever using conventional cigarettes, according to a 2018 report by the National Academies of Sciences, Engineering, and Medicine [7]. In addition, as social media has become an emerging channel for information diffusion, JUUL-related discussions on platforms such as Twitter and Reddit have also experienced a sharp increase since 2015 [8,9]. Thus, this sharp increase in teen JUUL usage, and the potential health impacts, has raised great public health concerns.

With the rapid increase in JUUL use, there have been a few studies about JUUL. Several studies have been conducted to investigate the prevalence of JUUL use among adolescents [10], youth [11,12], and college students [13]. Moran et al studied sources of awareness of JUUL e-cigarettes in 2 surveys of American adults [14]. Willett et al conducted a national Web-based survey to investigate youth and young adults’ knowledge, attitude, and use of JUUL [3]. Recently, Russel et al conducted a Web-based survey to assess transitions in cigarette smoking associated with the use of JUUL vaping device among 18,799 adults in the United States. [15]. Laestadius et al characterized the sale of JUUL products on eBay before a Food and Drug Administration (FDA) request to remove them, documented the impact of the request, and explored how eBay vendors bypassed FDA’s effort [16]. In addition, social media data have also been used to study JUUL-related communications and their impact. Huang et al studied the extent of JUUL’s growth and its marketing strategies using retail data form Nielsen and social media data from Twitter, Instagram, and YouTube [8]. JUUL-related tweets have been used to understand the public’s early experiences with JUUL [17], JUUL information sharing among adolescents [18], and identified patterns of communication around JUUL use and users on Twitter [19].

### Objective

Reddit has been extensively used for e-cigarette studies, which shows its value in timely public health surveillance. For example, Zhan et al identified and analyzed topics related to e-cigarettes from multiple social media platforms, including Twitter, Reddit, and JuiceDB [20]. Kavuluru et al described observations and analysis of recent JUUL-related messages on Twitter, Reddit, and in traditional media [9]. Brett et al used Reddit threads to understand reasons of JUUL use and public attitudes [21]. However, little research has been conducted on the age distribution, geographic distribution, approaches to buying JUUL, and flavor preferences pertaining to underage JUUL users. Until early January 2018, a subreddit entitled *UnderageJuul* existed, where these and other topics related to use of JUUL by youth were discussed. It was discontinued and removed by Reddit. Most casual users and analysts of Reddit thus no longer had access to the conversations in the UnderageJuul subreddit as of that date. However, we obtained part of the posting and user information of this subreddit for analyses pertaining to user age and location distributions, as well as self-reported usage patterns of JUUL. This study was aimed to inform the tobacco control community and those responsible for tobacco regulatory decision making as well as demonstrate the value of Reddit data for Web-based surveillance of public health issues.

## Methods

### Data Collection

Reddit data were collected from pushshift.io [22], which is a website that stores all publicly available Reddit threads and comments. Data were collected from 716 threads and 2935 comments from the subreddit UnderageJuul by the application programming interface (API) of this website. Note that these data were publicly accessible on Reddit and that no personally identifiable information is included in this study. The dataset is publicly available per request.

The first thread on the *UnderageJuul* subreddit was published on July 9, 2017, and the last was published on January 7, 2018, the day just before this subreddit was removed by Reddit. There were 844 unique users who posted threads and comments during this time period.

### Data Analysis

This dataset was analyzed from 3 perspectives. First, age and location distributions described characteristics of the Redditors and provided details describing who they were. Second, because some of these Redditors were self-reported underage JUUL users, we examined how they acquired JUUL products. Third, we focused on a specific use pattern, JUUL flavor choice, and analyzed the popularity of different JUUL flavors among these Redditors. For the analyses of these 3 perspectives, we used regular expression-enhanced keyword search, which is a method for locating specific character strings embedded in text [20], to determine the most relevant threads and comments. Python is used for these analyses.

First, age and geographic distribution of *UnderageJuul* users was summarized. Threads or comments containing numerical numbers or keywords *teen*, *twenty*, *thirty*, *forty*, *fifty*, *sixty*, *seventy*, *eighty*, and *ninety* were selected for further manual examination. If the thread or comment author explicitly mentioned that “I am # years old” or used similar statements, we assigned an age label to the Redditor. This age information was further aggregated to an age distribution table. Then, we analyzed the subreddit preferences of these Redditors. For each of them, we identified a list of subreddits they had posted to. Then, the cumulative occurrence of subreddits of all UnderageJuul Redditors was calculated.

To assess the geographic distribution of *UnderageJuul* Redditors, only locations that were explicitly mentioned were included. For example, location information could be a state, an area (eg, *bay area*), a county, a city, or a zip code. These locations were manually extracted by examining the content of the threads. The extracted text location information such as *Bergen county* were converted to a standard form *Bergen County, New Jersey, United States of America* by using the Python Package GeoPy [23]. The standardized location information was then used to generate the corresponding latitude and longitude, which were further fed into a Google Maps plot.

Next, purchase approaches were analyzed by manually examining the content of threads. Purchase approaches discussed in *UnderageJuul* were classified into 7 categories. The categories were generated by an inductive category development method [24]. During this process, 2 authors individually reviewed threads consecutively and determined whether a thread contained purchase approach information. If so, based on the text information, the reviewer determined whether to assign the purchase approach to an existing category or to generate a new category. After reviewing 50% of the materials, the 2 reviewers summarized the categories and reached consensus on all categories. The coding then continued with the second half of the data; no new categories were generated during this second phase of coding. After determining the categories, the 2 reviewers independently labeled the whole dataset; 93.7% (671/716) of threads had the same labeling. The Cohen kappa was 0.906, which is considered a good agreement. The 2 reviewers reached consensus for all records after discussion.

Owing to concerns about the potential that specific JUUL flavors might increase use by and appeal to youth, we studied the flavor distribution among *UnderageJuul* threads. We identified different flavor types from the official JUUL website and counted the occurrence of these flavors in the collected threads and comments by using the regular expression search.

## Results

The age distribution for the *UnderageJUUL* subreddit is shown in Table 1. We counted a Reddit user in the general categories of less than 18 years or over 21 years, if the user stated in their threads or comments that they were under 18 or greater than 21 years old; 67 out of 844 (7.9%) *UnderageJuul* Redditors reported their age in the threads or comments.

We analyzed the subreddit preferences of all *UnderageJuul* Redditors. Among 844 *UnderageJuul* Redditors, 437 had also posted threads on the *JUUL* subreddit, which is the general discussion subreddit of JUUL products. The other 9 subreddits in the top 10 most popular with *UnderageJUUL* users were as follows: *AskReddit* (general Q&A, n=250), *trees* (marijuana, n=145), *FortNiteBR* (computer game, n=125), *Drugs* (n=123), *pics* (interesting pictures sharing, n=106), *mildlyinteresting* (general discussion, n=103), *Showerthoughts* (general discussion, n=102), *teenagers* (n=96), and *funny* (general discussion, n=86). We also provided the full list of subreddit interests in Multimedia Appendix 1.

**Figure 1:**
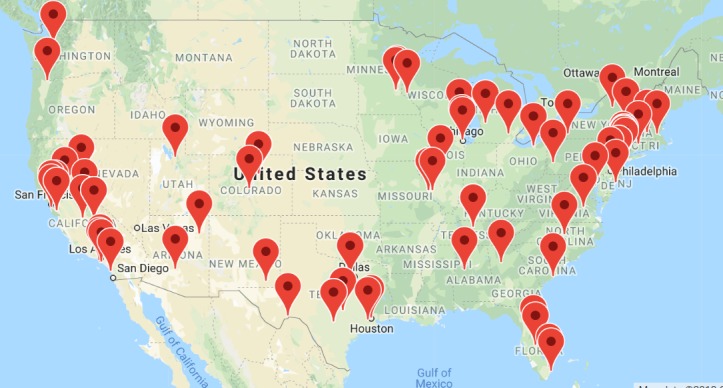
Explicitly mentioned location distributions.

We extracted 74 explicitly mentioned locations from the thread text. We summarized the mentioned locations and labeled them on Google Maps API, as shown on Figure 1. Among these locations, New York City and the Bay Area were the most mentioned areas. The detailed location count summary to each state can be found in Multimedia Appendix 2.

**Table 1:** The extracted age distribution of the *UnderageJuul* Redditors.

Age (years)	Count (n=67), n (%)
<18^a^	5 (7)
13	2 (3)
14	2 (3)
15	9 (13)
16	10 (15)
17	12 (18)
18	17 (25)
19	4 (6)
20	2 (3)
21	1 (1)
23	1 (1)
>21^a^	2 (3)

^a^These are broad categories of age where the user did not give a specific age but stated generally that they were under age (<18) or a legal adult (>21).

Next, we analyzed the approaches that were used to purchase JUUL by *UnderageJuul* users. By examining purchase requests and completed transactions in detail, we observed that there were 7 approaches that users employed to buy JUUL. These approaches are listed as follows:

1. JUUL’s official website (n=34): Although JUUL changed their age policy and raised the minimum purchase age to 21 years in August, 2018 [25], JUUL required its users to be at least 18 years to order from its official website when the dataset was collected, but some *UnderageJuul* Redditors (n=24) used identity documents (IDs), date of birth, or credit cards of parents or friends to buy JUUL products. Using fake IDs (n=3) was another approach to buy JUUL products from the JUUL website. Some Redditors (n=7) were selling verified accounts on JUUL’s official website, which could be used to order JUUL products as well.
2. Other vaping-related websites (n=13): Some of the other vaping-related websites sold JUUL devices and pods without age verification. Some *UnderageJuul* Redditors exchanged information about these websites. For example, 1 user asked if a particular website does an age check for buying JUUL products. Currently, this website does not sell JUUL, but it does sell e-cigarette products without age verifications. Other sites were shared, including 1 selling JUUL that has since been removed by law enforcement.
3. Peer-to-peer purchase and shipping (n=181): Some *UnderageJuul* Redditors bought JUUL products from other Redditors. The buyer usually sent money by PayPal and then the seller shipped the products to the buyer’s address. A noteworthy observation was that some of the Redditors claimed they were scammed. For example, 1 user said in a thread, “don’t trust ***, he scammed me and will scam you.”
4. Through vendors from Amazon and eBay (n=27): This was another case of Web-based purchase and shipping. The vendor had an Amazon or eBay selling page from which the buyers purchased JUUL products. For example, 1 user shared the experience that he or she bought a JUUL from eBay and had it shipped to a mailbox of a for-sale house.
5. Face-to-face transactions (n=22): Some tried to meet up with other Reddit users in the same city and then finished transactions face to face.
6. Replacement codes of JUUL (n=47): Some bought JUUL products by using JUUL replacement codes or warranty serial numbers shared by other Redditors. The replacement codes or warranty serial numbers are provided by the JUUL company and can be used to get a brand new JUUL devices as a replacement. For example, 1 user mentioned that he wanted to sell a replacement code for $30 that could be used for a free basic kit.
7. Local stores without checking IDs (n=35): Some *UnderageJuul* Redditors did not try to buy JUUL products from other individuals. Instead, they inquired about vape shops or smoke shops in certain locations that sold JUUL but did not check IDs. For example, 1 user asked for vaping stores in NYC that did not check ID card of the buyers.

Finally, we analyzed the flavors mentioned in the subreddit. There were 7 flavors from JUUL’s official website: mango, cucumber, mint, menthol, tobacco, fruit medley, and crème brûlée. We found that mango was the most mentioned flavor (n=38). Mint (n=22) and cucumber (n=21) were popular as well. The other 4 flavors, including fruit medley (n=6), crème brûlée (n=6), menthol (n=5), and tobacco (n=4), were not frequently mentioned.

## Discussion

In this study, we obtained the following main findings pertaining to demographics, methods of access, product characteristics, and use patterns of underage JUUL use. First, most threads or comments did not mention specific age, but ages ranged from 13 years to greater than 21 years in those that did. Second, mango, mint, and cucumber were the most popular among the 7 flavors from JUUL’s official website, and 336 subreddit *UnderageJuul* threads mentioned 7 discreet approaches to circumvent relevant legal regulations to get JUUL products, the most common of which was purchasing JUUL from other Reddit users (n=181). Third, almost half of the *UnderageJuul* users (389/844, 46.1%) also participated in discussions on the main *JUUL* subreddit and were seeking information across multiple Reddit forums. Finally, most (64/74, 86%) posters were from large metropolitan areas defined by the United States Office of Management and Budget [26]. These findings could help public health community and policy makers understand the current situation of underage JUUL use and provide them with insights on how to leverage social media for timely surveillance of emerging public health issues.

We found that only a small number of Redditors (67/844, 7.9%) reported their ages in a subreddit for underage JUUL users. After the FDA’s deeming rule, the minimum age to purchase e-cigarettes for all states in the United States is 18 years. If we set 18 years as the legal age threshold, 60% (40/67) of the Reddit users in Table 1 were underage JUUL users. Alabama, Alaska, and Utah have set 19 years as the legal age to buy e-cigarettes and vaping products. In California, Hawaii, Maine, Oregon, and New Jersey, the legal age is 21 years. There are also more than 330 states, cities, and counties that have raised the minimum age to 21 years. Thus, if we raise the legal age threshold to 21 years, 94% (63/67) of the Reddit users in Table 1 were underaged. As purchasing JUUL is not legal for underage Redditors, they were more likely to avoid discussing their ages or to report fake ages. Therefore, the age distribution we obtained from explicit mentions was biased. The actual age distribution could probably be even younger. Given subreddit *UnderageJuul* was an active forum oriented for underage JUUL users before it was banned, it is still a valuable data source for analyzing underage JUUL users and their usage patterns.

We found that there was an overlap of users between the *JUUL* subreddit and the *UnderageJuul* subreddit. Specifically, 46.1% (389/844) of UnderageJuul Redditors also participated in discussions on the *JUUL* subreddit. Given the fact that most of the JUUL-related discussions on Reddit are on the *JUUL* subreddit, it is reasonable to infer that even though the *UnderageJuul* subreddit was removed by Reddit, underage JUUL users still have other forums for discussing JUUL with other users. This finding indicates that public health interventions, such as social media campaigns, are needed to target underage e-cigarette users.

We also analyzed the interests of the *UnderageJuul* users. These interests were extracted from their Reddit profiles and indicated some of these users contributed to teenager-related topics on Reddit. This finding suggests that some *UnderageJuul* Redditors have a high probability to be real underage JUUL users, though most of them did not report their ages in the *UnderageJuul* subreddit.

This study found that most of the geographic locations mentioned in threads were from large cities in the United States. In particular, New York City and the Bay Area were the most mentioned areas. The location information was collected via user-posted JUUL Web-based vendors, shipping information, or simply inquiring about local JUUL stores. As many of the inquiries were about local vape or smoke shops without age verification, the findings suggest that social media could be a useful tool for federal and state regulatory authorities to use for assessing whether vape or smoke shops are meeting regulatory requirements pertaining to e-cigarette use, especially in large cities.

Our study found that mango, mint, and cucumber flavors were more popular than other JUUL flavors among youth and young adult Reddit users, which is similar to previous findings that mint, cucumber, and mango JUUL flavors were most mentioned in tweets [9]. From previous research about e-cigarette flavors [27], strawberry was the most mentioned fruit flavor for other e-cigarette products. Instead, mango or cucumber flavors were hardly mentioned. However, these 2 flavors turned out to be successful for JUUL, suggesting that more effort is needed to understand if some flavors are more appealing to underage e-cigarette users or if a product’s appeal is more important than a specific flavor availability. Tobacco flavor was widely mentioned in previous e-cigarette studies, but it was not popular in youth and young adults. One possible reason is that many of the underage JUUL users are nonsmokers, so the tobacco flavor is not appealing. The finding suggests that more research is needed for studying the impact of e-cigarette flavors on underage e-cigarette use.

Our study found that users of the *UnderageJuul* subreddit used different discreet approaches to circumvent relevant legal regulations to get JUUL. Some of these approaches (eg, purchase via eBay) are also reported in previous studies [9,16]. This study and the findings contribute to the literature by providing a detailed category dictionary for purchase approaches. On the basis of the well-established coding method, we developed 7 different categories for purchase approaches [24]. Our finding suggests that more regulation is needed to make sure that online or offline e-cigarette vendors strictly perform age verification and that legal e-cigarette users cannot provide ways of buying e-cigarettes to teenagers. In addition, parents may need to assess their teenagers’ access to credit cards and IDs of legal adults.

Note that JUUL has established their age policy and required the minimum purchase age on their official website to be 21 years since August 2018. The JUUL product replacement request is also subject to the age verification. However, the identified 7 categories of workaround purchase approaches still work. Ordering the products or replacements from the official website just needs a fake ID or a preverified account. Not to mention those approaches provided by third parties such as eBay or PayPal. As the prevalence of teen JUUL use [28] and the high conversion rate from e-cigarette users to smokers [29], we call for more research and effort on this domain and take action to monitor and control these approaches.

Although this Web-based forum facilitates the JUUL transactions under the table, we still observed Redditors who tried to persuade teenagers not to start the vaping habit or not to get addicted to nicotine, especially for those who were extremely young, such as 13- or 14-year-old teens. This phenomenon represented the goodwill of the community. These comments also create additional questions about the specific population using *UnderageJuul*. Were those who made comments recommending against the use of ENDS products adults or youth? If they were adults, who are they, and why are they contributing to this particular subreddit? We cannot answer these questions given the limited information on this online forum, but we believe it is a promising future research direction to study the community and environment of underage e-cigarette users.

This study has some limitations. First, we could not verify if the self-reported ages in the data were real or fake owing to the anonymity of Reddit users. Most of these ages were general, and these Reddit users often stated that they were underage or legal adults. However, as this subreddit was specifically created for discussion of underage JUUL users, we think the mentioned ages were fairly reliable. In addition, the number of mentioned ages was relatively small compared with the number of the subreddit users, and only a small number of users shared an age. Second, the location distribution in this study faces a similar limitation as the age distribution, because we cannot get the exact addresses of Redditors from Reddit. In this study, we did not distinguish buyers’ locations and sellers’ locations, as we focused on the locations related to JUUL transactions. Third, the number of different flavors mentioned in the data was relatively small, but we can see their relative popularity among these Redditors. Fourth, we could not determine whether some messages were posted by e-cigarette store owners, others who have an interest in selling e-cigarettes, or even robots because the demographics of Reddit users were not available.

Despite the above limitations, the findings of this study have some important implications in terms of research and policy. It is clear that discussions about preferred flavors was 1 reason that users used the UnderageJuul subreddit and also that many users used this venue to explore ways to obtain access to JUUL. At the same time, in the 6 months that this venue existed, it was not heavily used. However, because about half of the *UnderageJuul* users also used the *JUUL* subreddit, there are alternative venues whereby youth could obtain information on flavors and methods to obtain JUUL. Our analysis of the subreddit for underage JUUL users suggests that social media data are a valuable data source for rapid public health surveillance, especially for emerging products such as JUUL, as the popularity of such products is often accompanied by discussions and promotions of the products on social media [8]. There are several limitations of these data, and they should not be the only source of information, but they provide a unique and potentially real-time source of data to better understand behavioral intentions, potential demographic variables, purchase behaviors, social networks (which were not assessed in this analysis), temporal changes across a variety of variables, and other data that could be valuable to the public health, research, and regulatory communities.

In addition, our findings indicate that social media could be a valuable surveillance tool for federal and state authorities responsible for regulating stores that sell e-cigarettes. Each state in the United States has a robust, FDA-funded process for tracking and regulating such stores, and social media data could be used to identify stores where additional assessment or observation and enforcement may be needed. When used along with other sources of data, social media data can point to emerging issues that need to be addressed in more depth.

## Appendix

Multimedia Appendix 1 Subreddit interest.

Multimedia Appendix 2 Location count.

## Abbreviations

API: application programming interface
FDA: Food and Drug Administration
ID: identity document
USB: universal serial bus
