# Supplementary material for: Underage JUUL Use Patterns: Content Analysis of Reddit Messages
Source: J Med Internet Res. 2019 Sep 9;21(9):e13038. doi: 10.2196/13038 (PMC6786857; doi:10.2196/13038)
Supplement: Multimedia Appendix 1 [file jmir_v21i9e13038_app1.pdf]

Appendix 1 UnderageJuul Redditors Subreddit Interest

juul	437
AskReddit	250
trees	145
FortNiteBR	124
Drugs	123
pics	106
Showerthoughts	102
mildlyinteresting	101
teenagers	96
funny	86
aww	82
gaming	81
FashionReps	80
streetwear	75
electronic_cigarette	74
Sneakers	65
gifs	64
supremeclothing	64
pcmasterrace	64
Vaping	60
hiphopheads	60
dankmemes	60
news	58
videos	57
explainlikeimfive	50
weed	49
todayilearned	49
BlackPeopleTwitter	46
thanosdidnothingwrong	45
Jokes	44
LSD	44
AskOuija	43
me_irl	43
Repsneakers	43
RoastMe	43
jailbreak	41
CircleofTrust	39
The_Donald	39
interestingasfuck	39
buildapc	39
GlobalOffensive	39
trashy	39
sports	35

OldSchoolCool	34
xboxone	33
worldnews	33
Music	33
starterpacks	33
NoStupidQuestions	32
cars	31
tifu	31
legaladvice	30
Bitcoin	30
personalfinance	29
food	29
saplings	29
IAmA	29
mildlyinfuriating	28
apple	28
DesignerReps	28
movies	28
ecigclassifieds	28
iphone	27
Fitness	27
Tinder	27
EarthPorn	27
oilpen	27
memes	27
ApplyingToCollege	26
depression	26
whatisthisthing	26
hardwareswap	26
Advice	26
nba	26
askscience	25
Overwatch	25
LifeProTips	25
FashionRepsBST	25
thatHappened	25
XXXTENTACION	24
television	24
madlads	24
WTF	24
DeepFriedMemes	24
iamverysmart	24
oddlysatisfying	24
Rainbow6	23
rickandmorty	23

Kanye	23
nottheonion	23
CrappyDesign	23
tipofmytongue	23
Phix	22
vaporents	22
PUBATTLEGROUNDS	22
MemeEconomy	21
Sat	21
pokemongo	21
CringeAnarchy	21
leagueoflegends	21
techsupport	21
science	21
ImGoingToHellForThis	21
sneakermarket	20
relationships	20
confession	20
Waxpen	19
RepTime	19
DrugStashes	19
ComedyCemetery	19
watchpeopledie	19
assholedesign	19
Cigarettes	19
USPS	18
AskDocs	18
politics	18
h3h3productions	18
StonerEngineering	18
WWII	18
FellowKids	18
slavelabour	18
nfl	18
giftcardexchange	17
opiates	17
Supreme	17
G59	17
DunderMifflin	17
hmmm	17
greentext	17
softwaregore	17
NintendoSwitch	16
woahdude	16
SuorinAir	16

benzodiazepines	16
CryptoCurrency	16
4chan	16
im14andthisisdeep	16
BikiniBottomTwitter	16
FORTnITE	16
DarkNetMarketsNoobs	16
darkjokes	16
PUBG	16
airsoft	16
researchchemicals	16
iamverybadass	16
Whatcouldgowrong	16
dataisbeautiful	16
malefashionadvice	16
GlobalOffensiveTrade	16
sex	15
gtaonline	15
NoFap	15
baseball	15
wholesomememes	15
pokemon	15
technology	15
soccer	15
space	15
travisscott	15
WhitePeopleTwitter	15
insanepeoplefacebook	15
Watches	14
Suorin	14
MechanicalKeyboards	14
UnethicalLifeProTips	14
AdviceAnimals	14
EDC	14
nosleep	14
APStudents	14
PalaceClothing	14
CBD	14
LateStageCapitalism	14
LilPeep	14
dxm	14
shrooms	14
MadeMeSmile	14
TwoXChromosomes	13
Piracy	13

sadcringe	13
Art	13
CODZombies	13
justneckbeardthings	13
PoliticalHumor	13
instant_regret	13
listentothis	13
4PanelCringe	13
CallOfDuty	13
WritingPrompts	13
Unexpected	13
AskMen	13
PrequelMemes	13
ADHD	13
Vaping101	13
photoshopbattles	13
streetwearstartup	12
malehairadvice	12
ClashRoyale	12
ClashOfClans	12
summonerschool	12
lacrosse	12
battlestations	12
SkincareAddiction	12
drunk	12
playrust	12
GetMotivated	12
Android	12
ACT	12
wow	12
MechanicAdvice	12
IdiotsInCars	12
drugscirclejerk	12
snapchat	12
unpopularopinion	12
Steam	12
Ice_Poseidon	12
PublicFreakout	12
headphones	11
conspiracy	11
ChoosingBeggars	11
meirl	11
MaddenMobileForums	11
hearthstone	11
cringe	11

TooAfraidToAsk	11
Badfaketexts	11
rap	11
keto	11
adderall	11
IASIP	11
Frat	11
atheism	11
litecoin	11
csgo	11
BeAmazed	11
wallstreetbets	11
NBA2k	11
DestinyTheGame	11
copypasta	11
OutOfTheLoop	11
sneakerbots	10
DippingTobacco	10
VapePorn	10
ATBGE	10
woooosh	10
NatureIsFuckingLit	10
marvelstudios	10
chanceme	10
blackmirror	10
Stims	10
GameSale	10
pcgaming	10
whatcarshouldIbuy	10
Perfectfit	10
GTAV	10
niceguys	10
lgbt	10
VaporwaveAesthetics	10
FortniteBattleRoyale	10
EntExchange	10
youtube	10
natureismetal	10
fakehistoryporn	10
findareddit	10
SnapLenses	9
inthesoulstone	9
RocketLeagueExchange	9
FreeKarma4You	9
anime	9

deepweb	9
Dabs	9
college	9
Conservative	9
Damnthatsinteresting	9
leaves	9
quityourbullshit	9
Fallout	9
RocketLeague	9
iamatotalpieceofshit	9
FIFA	9
EDM	9
Anxiety	9
MDMA	9
shoebots	9
buildapcsales	9
blackops3	9
creepy	9
MaddenUltimateTeam	9
crappyoffbrands	9
OopsDidntMeanTo	9
beermoney	9
cursedimages	9
brockhampton	9
subaru	9
Futurology	9
Minecraft	9
BMW	9
CoDCompetitive	8
motorcycles	8
LivestreamFail	8
drugsarebeautiful	8
tattoos	8
pyrocynical	8
phenibut	8
cocaine	8
youtubehaiku	8
confessions	8
highschool	8
cringepics	8
notinteresting	8
nostalgia	8
bapeheads	8
Smite	8
confusing_perspective	8

books	8
garlicoin	8
RepVouch	8
Memes_Of_The_Dank	8
2007scape	8
relationship_advice	8
Nootropics	8
smashbros	8
PewdiepieSubmissions	8
antisocialsocialclub	8
dontdeadopeninside	8
rage	8
Animemes	8
oldpeoplefacebook	8
PS4	8
circlejerk	8
HomeworkHelp	8
NYCtrees	8
gatekeeping	8
casualiama	8
shameless	8
gameswap	8
UpliftingNews	8
RobinHood	8
shittyfoodporn	8
GothBoiClique	8
ProgrammerHumor	8
AMA	8
Wellthatsucks	8
Eyebleach	8
gonewild	7
halo	7
facepalm	7
nevertellmetheodds	7
TumblrInAction	7
iOSthemes	7
Roadcam	7
carporn	7
engrish	7
rarepuppers	7
loseit	7
PhotoshopRequest	7
JuulPod	7
fuckthepopulation	7
cats	7

RotMG	7
newjersey	7
LGBTeens	7
PornhubComments	7
GCXRep	7
bonehurtingjuice	7
CasualConversation	7
indianpeoplefacebook	7
2meirl4meirl	7
EscapefromTarkov	7
applehelp	7
investing	7
PUBGMobile	7
ThriftStoreHauls	7
playboicarti	7
farcry	7
reactiongifs	7
Blackops4	7
golf	7
FrankOcean	7
Libertarian	7
okbuddyretard	7
StarWars	7
Documentaries	7
Flipping	7
dadjokes	7
medical	7
tipofmypenis	7
RecruitCS	7
Psychedelics	7
GarlicBreadMemes	7
blackmagicfuckery	7
creepyPMs	7
longboarding	7
CannabisExtracts	7
learnprogramming	6
Instagram	6
OFWGKTA	6
Justrolledintotheshop	6
mildlypenis	6
teenagersnew	6
CatsStandingUp	6
WatchPeopleDieInside	6
hookah	6
Jeep	6

HowToHack	6
NameThatSong	6
Dreams	6
Ooer	6
iPhoneX	6
KidsAreFuckingStupid	6
SuicideWatch	6
betterCallSaul	6
AnimalsBeingBros	6
ihavesex	6
redditgetsdrawn	6
chinaglass	6
laptops	6
aves	6
popping	6
IsItBullshit	6
paypal	6
Rateme	6
lego	6
stocks	6
DDLC	6
ass	6
DoesAnybodyElse	6
gratefuldead	6
Fishing	6
progresspics	6
buildapcforme	6
WouldYouRather	6
RandomKindness	6
liluzivert	6
NoMansSkyTheGame	6
Justfuckmyshitup	6
FortniteCompetitive	6
NotMyJob	6
GalaxyS8	6
Marijuana	6
HumansBeingBros	6
Lollapalooza	6
donaldglover	6
BetterEveryLoop	6
ChildrenFallingOver	6
ArtOfRolling	6
HomeNetworking	6
whatsthisbug	6
Vape_Porn	6

Greekgodx	6
Ebay	6
zedmains	6
StarWarsBattlefront	6
happy	6
battlefield_one	6
vapeitforward	6
NotHowDrugsWork	6
morbidquestions	6
history	6
thalassophobia	6
FiftyFifty	6
treedibles	6
bodybuilding	6
Accutane	6
Idubbbz	6
dating_advice	6
offmychest	6
outrun	6
kratom	6
shittyaskscience	6
SuggestALaptop	6
HeistTeams	6
Bossfight	6
nyc	6
onions	6
hittableFaces	6
IllegalLifeProTips	6
sportsbook	6
ShittyLifeProTips	6
Eminem	6
airsoftmarket	6
tmobile	6
theydidthemath	6
hacking	5
3Dprinting	5
freebies	5
MurderedByWords	5
spotify	5
bicycling	5
DIY	5
randomactsofcsgo	5
sideloaded	5
antiMLM	5
Shitty_Car_Mods	5

PandR	5
governorsball	5
magicTCG	5
alcohol	5
cigars	5
Petioles	5
FlashTV	5
place	5
chicago	5
seduction	5
appleswap	5
TrueOffMyChest	5
destiny2	5
trashyboners	5
BoJackHorseman	5
sbubby	5
Entrepreneur	5
instantkarma	5
loltyler1	5
LucidDreaming	5
fantasyfootball	5
skyrim	5
OSHA	5
trap	5
AppleWatch	5
VPN	5
powerwashingporn	5
tippytaps	5
borrow	5
ethereum	5
Frugal	5
TheLastAirbender	5
gameofthrones	5
computers	5
Vans	5
rant	5
Naruto	5
MaliciousCompliance	5
KarmaConspiracy	5
apolloapp	5
itookapicture	5
evilbuildings	5
amazon	5
bostonceltics	5
SteamGameSwap	5

FilthyFrank	5
electronicmusic	5
WhatsWrongWithYourDog	5
bindingofisaac	5
KendrickLamar	5
nononono	5
redditbay	5
eagles	5
JusticeServed	5
CrazyIdeas	5
translator	5
Seaofthieves	5
makinghiphop	5
FidgetSpinners	5
mechmarket	5
urbanexploration	5
beetlejuicing	5
mac	5
tumblr	5
therewasanattempt	5
asktrp	5
jobs	5
btc	5
gadgets	5
Logic_301	5
juulrefill	5
PeopleFuckingDying	5
FloridaMan	5
Psychonaut	5
westworld	5
Patriots	5
Games	5
mflb	5
doordash	5
flatbushzombies	5
netflix	5
NiceHash	5
raisedbynarcissists	5
HelpMeFind	5
snowboarding	5
guns	5
drums	4
ShouldIbuythisgame	4
comedyheaven	4
CFB	4

IBO	4
csgotrade	4
ft86	4
HipHopImages	4
StrangerThings	4
moviepass	4
AmItheAsshole	4
Grailed	4
CatastrophicFailure	4
see	4
creepypasta	4
brooklynninenine	4
Doom	4
lostredditors	4
SweatyPalms	4
FL_Studio	4
microgrowery	4
forhonor	4
PUBGXboxOne	4
torrents	4
verizon	4
smoobypost	4
MakeupAddiction	4
Gamingcirclejerk	4
JizzedToThis	4
MLS	4
skiing	4
teslamotors	4
nocontextpics	4
trebuchetmemes	4
europe	4
totallynotrobots	4
Warframe	4
rotmgtradingpost	4
diablo3	4
MakeNewFriendsHere	4
Target	4
Favors	4
StreetwearSales	4
Dentistry	4
RandomActsOfGaming	4
CHICubs	4
amiugly	4
uselessredcircle	4
OnePiece	4

AskNYC	4
Amd	4
UnresolvedMysteries	4
Windows10	4
nonononoyes	4
iOSBeta	4
Blink182	4
Mustang	4
thewalkingdead	4
breakingbad	4
ketamine	4
2healthbars	4
HistoryMemes	4
1P_LSD	4
pebble	4
MMA	4
GamePhysics	4
techsupportgore	4
millionairemakers	4
Cartalk	4
AskHistorians	4
sixers	4
MandelaEffect	4
CryptoMarkets	4
bigdickproblems	4
Braincels	4
lifehacks	4
Miata	4
snakes	4
paintball	4
JoeRogan	4
Supreme_NYC	4
caps	4
Maplestory	4
Cameras	4
trailerparkboys	4
CozyPlaces	4
NavyBlazer	4
NYYankees	4
Surface	4
fountainpens	4
GameTrade	4
highdeas	4
Ripple	4
GooglePixel	4

Twitch	4
coins	4
popheads	4
dank_meme	4
wicked_edge	4
YasuoMains	4
javahelp	4
PAXvapor	4
boottoobig	4
GrandTheftAutoV	4
NewTubers	4
Borderlands2	4
GamersRiseUp	4
ScottishPeopleTwitter	4
gtaglitches	4
ios	4
holdmycosmo	4
AccidentalWesAnderson	4
forbiddensnacks	4
CallOfDutyWorldWarTwo	4
holdmybeer	4
Fantasy_Football	4
boston	4
iphonehelp	4
watercooling	4
delusionalcraigslist	4
postmates	4
socialanxiety	4
Paladins	4
AccidentalRacism	4
Brawlhalla	4
MapPorn	4
AwesomeCarMods	4
Dell	4
Whatisthis	4
Metalcore	4
RoastMyCar	4
ChanceTheRapper	4
Graffiti	4
DMT	4
malefashion	4
help	4
longisland	4
audiophile	4
afinil	4

MouseReview	4
bisexual	4
Pareidolia	4
MarvelStudiosSpoilers	4
DotA2	4
CollegeBasketball	4
educationalgifs	4
discordapp	4
communism	4
Autos	4
SampleSize	4
disneyvacation	4
HaloOnline	4
TalesFromRetail	4
photography	4
samsung	4
buildmeapc	4
3dshacks	4
macbook	4
Brawlstars	4
hockey	4
glassheads	4
washingtondc	4
Bestbuy	4
blunderyears	4
OverwatchUniversity	4
projectcar	4
Knife_Swap	4
Aquariums	4
The_Mueller	4
AbandonedPorn	4
shareyourmusic	3
linuxquestions	3
mechanical_gifs	3
pillhead	3
Christianity	3
gifsthatkeepongiving	3
PocketMortys	3
SBU	3
iosgaming	3
StackAdvice	3
albania	3
Anarcho_Capitalism	3
195	3
answers	3

WhatIsThisPainting	3
redsox	3
Accounting	3
GamingMarket	3
DirtySnapchat	3
csgobetting	3
networking	3
AlphaBay	3
pens	3
MealPrepSunday	3
terriblefacebookmemes	3
alteredcarbon	3
Flexicas	3
torrentlinks	3
PKA	3
DiWHY	3
ShowerOrange	3
Boruto	3
Assistance	3
wownoob	3
disenchantment	3
Money	3
DataHoarder	3
UberEATS	3
UMD	3
LosAngeles	3
CCW	3
writing	3
vaxxhappened	3
running	3
povertyfinance	3
UNBGBBIIVCHIDCTIICBG	3
MW2	3
ShadowBan	3
TheRedPill	3
churning	3
VirginiaTech	3
BreakUps	3
CoinBase	3
im14andthisisfunny	3
PuzzleAndDragons	3
GiftofGames	3
venmo	3
Jazz	3
flying	3

audio	3
yeezys	3
airpods	3
HighQualityGifs	3
gardening	3
Lyft	3
Lifeguards	3
exchristian	3
lonely	3
GTA	3
hamptonbrandon	3
Csgohacks	3
scooters	3
succulents	3
thick	3
whatsthisplant	3
woweconomy	3
DIY_eJuice	3
WeWantPlates	3
BSA	3
deadbydaylight	3
highcandy	3
dankvideos	3
ToiletPaperUSA	3
MaleFashionMarket	3
AntiJokes	3
Debate	3
LofiHipHop	3
MkeBucks	3
navy	3
Cubers	3
fastfood	3
TheOA	3
titanfall	3
AskPhysics	3
GifSound	3
tf2	3
travel	3
BitcoinMining	3
malelivingspace	3
SlumpGod	3
hockeyplayers	3
socialmedia	3
badtattoos	3
TripSit	3

DAE	3
arrow	3
emojipasta	3
wholesomebpt	3
sad	3
GolfGTI	3
addiction	3
Comcast_Xfinity	3
wholesomegifs	3
socialskills	3
GameDeals	3
BattlefrontTWO	3
forwardsfromgrandma	3
SwagBucks	3
BuyItForLife	3
FortniteFashion	3
photocritique	3
Disneyland	3
warpedtour	3
dayz	3
Hacking_Tutorials	3
bravefrontier	3
NobodyAsked	3
hardware	3
jailbreakdevelopers	3
changemyview	3
Marvel	3
glitch_art	3
comics	3
BitMarket	3
BokuNoHeroAcademia	3
mlb	3
trippinthroughtime	3
whatsthissnake	3
suspiciousquotes	3
surfing	3
PoliticalDiscussion	3
SpidermanPS4	3
replications	3
h1z1	3
perfectloops	3
gainit	3
SandersForPresident	3
supergirlTV	3
Guitar	3

FoodPorn	3
customhearthstone	3
ThreadGames	3
ElsaGate	3
SwordOrSheath	3
NHLHUT	3
cpp_questions	3
FifaCareers	3
TwoRedditorsOneCup	3
NJTech	3
FortnitePS4	3
shopify	3
Calligraphy	3
Seattle	3
frugalmalefashion	3
ProRevenge	3
vita	3
THE_PACK	3
SLRep	3
Coffee	3
orangeisthenewblack	3
Vive	3
bloodborne	3
drugmemes	3
thedivision	3
sadboys	3
soylent	3
MosinNagant	3
AskWomen	3
BiggerThanYouThought	3
traaaaaaannnnnnnnnns	3
BostonBruins	3
Breath_of_the_Wild	3
Cyberpunk	3
PodMods	3
shittyama	3
NSFW411	3
short	3
PostMalone	3
cakeday	3
MeanJokes	3
AfterEffects	3
shittyrainbow6	3
PixelCarRacer	3
ShinyPokemon	3

tylerthecreator	3
lost	3
theocho	3
chairsunderwater	3
webdev	3
SuddenlyGay	3
KitchenConfidential	3
NewSkaters	3
Rivenmains	3
learnpython	3
SubsYouFellFor	3
woodworking	3
BartardStories	3
selfharm	3
vape_deals	3
bostontrees	3
savedyouaclick	3
toosoon	3
runescape	3
cheatatmathhomework	3
Barca	3
CampingandHiking	3
ableton	3
holdmyredbull	3
pcgamingtechsupport	3
edmproduction	3
ShadowBanned	3
computer_help	3
nvidia	3
ComedyNecrophilia	3
Retconned	3
highthoughts	3
ColorizedHistory	3
DeFranco	3
Hardcore	3
Battlefield	3
counterstrike	3
pinkfloyd	3
schizophrenia	3
NSFWFunny	3
self	3
Xenoblade_Chronicles	3
Meditation	3
SaltLakeCity	3
nintendo	3

ChiefKeef	3
gamingpc	3
ofcoursethatsathing	3
BoneAppleTea	3
lgg4	3
ethtrader	3
FunnyandSad	3
TheSilphRoad	3
SneakerMarketRefs	3
jobuds	3
surrealmemes	3
childfree	3
dpdr	3
fo4	3
Blep	3
nuzlocke	3
bestof	3
friendsafari	3
Vaporwave	3
boostedboards	3
ShroomID	3
awfuleyebrows	3
OffensiveMemes	3
ambien	3
Parenting	3
audioengineering	3
WeAreTheMusicMakers	3
overclocking	3
MortalKombat	3
SCP	3
DeepIntoYouTube	3
tipofmyjoystick	3
lean	3
CapitalismVSocialism	3
porn_gifs	3
PokemonGoSpoofing	3
Corsair	3
survivor	3
Watchexchange	2
tea	2
Gameboy	2
HouseOfCards	2
battlefield_4	2
metalgearsolid	2
RapLeaks	2

3DS	2
feetpics	2
Bass	2
newhampshire	2
WaltDisneyWorld	2
Hiphopcirclejerk	2
ComedyHitmen	2
chemistry	2
fasting	2
MacOS	2
BitcoinBeginners	2
TittyDrop	2
amathenedit	2
cursedcursedimages	2
ucf	2
bipolar	2
Rabbits	2
PornStars	2
Polaroid	2
MLBTheShow	2
RepLadies	2
GuitarHero	2
askcarsales	2
dubai	2
synthrecipes	2
Zippo	2
rotmgvouches	2
LSA	2
MovieDetails	2
androidapps	2
AdrenalinePorn	2
guitarcirclejerk	2
Dirtbikes	2
acting	2
harrypotter	2
InclusiveOr	2
vegan	2
holdmyfries	2
FortniteBRMeta	2
JUSTNOMIL	2
auburn	2
shittyreactiongifs	2
mcservers	2
wholesomeanimemes	2
ASU	2

AskThe_Donald	2
Drizzy	2
Beatmatch	2
lewronggeneration	2
HappyEmbarrassedGirls	2
vainglorygame	2
Vape_Reviews	2
sandiego	2
RepWatch	2
UIUC	2
feedthebeast	2
puns	2
ElderScrolls	2
DenzelCurry	2
web_design	2
tooktoomuch	2
microdosing	2
thegrandtour	2
BestOfStreamingVideo	2
IndianaUniversity	2
Scams	2
masterhacker	2
supermoto	2
migos	2
icocrypto	2
battlewagon	2
DesignPorn	2
MTVScream	2
earthbound	2
arcane	2
ReverseChanceMe	2
xTrill	2
SiliconValleyHBO	2
Chipotle	2
nathanforyou	2
ski	2
windows	2
Fuckthealtright	2
analog	2
PandoranRedCross	2
pornfree	2
circumcision	2
rule34	2
Random_Acts_Of_Pizza	2
AskTrumpSupporters	2

Drama	2
AndroidHelp	2
PhantomForces	2
minibikes	2
samuraijack	2
sunglasses	2
ffxiv	2
nunumains	2
Shave_Bazaar	2
KeanuBeingAwesome	2
Breadit	2
UnpopularOpinions	2
infj	2
ClashOfClansRecruit	2
OCD	2
pettyrevenge	2
askwomenadvice	2
marketing	2
couriersofreddit	2
Ratemeteen	2
Gear4Sale	2
Paranormal	2
Rengarmains	2
ask	2
Stance	2
Cuckold	2
Vape_Chat	2
JuulSupport	2
dropship	2
suboxone	2
hiphop	2
FortniteRoyaleLFG	2
Jeopardy	2
gunpolitics	2
Bitcoincash	2
JordanPeterson	2
talesfromcavesupport	2
pathofexile	2
Monstercat	2
MacMiller	2
TalesFromThePizzaGuy	2
CheggAnswerRequest	2
warriors	2
eu4	2
SneakerDeals	2

RLFashionAdvice	2
TalesFromYourServer	2
hqtrivia	2
AskBattlestations	2
wokekids	2
Celebs	2
fffffffuuuuuuuuuuuu	2
AskRedditAfterDark	2
CryptoKitties	2
knives	2
walmart	2
RedditInReddit	2
ultrawidemasterrace	2
Celica	2
CampFlognaw	2
ElectricSkateboarding	2
bose	2
ProtectAndServe	2
rush	2
FreeKarma4U	2
Dexter	2
Superbowl	2
Volvo	2
Tendies	2
MCSRep	2
adorableporn	2
thisismylifenow	2
NHL18	2
Astronomy	2
LootRoyale	2
SubOhmTanks	2
wifesharing	2
RealGirls	2
TOR	2
Sexsells	2
cubancigars	2
muacirclejerk	2
ufl	2
dontputyourdickinthat	2
Throwers	2
FixedGearBicycle	2
AstralProjection	2
manga	2
SquaredCircle	2
absolutelynotme_irl	2

steelers	2
HENTAI_GIF	2
HUTrep	2
minnesota	2
econhw	2
Sneks	2
dbz	2
regularcarreviews	2
TheGirlSurvivalGuide	2
Feminism	2
KarmaCourt	2
roosterteeth	2
GrandTheftAutoV_PC	2
namethatcar	2
Golfwang	2
pokemontrades	2
ploompax	2
blog	2
WatchItForThePlot	2
Detroit	2
porninfifteenseconds	2
StonerProTips	2
Charlotte	2
razer	2
darksouls3	2
KaynMains	2
Spiderman	2
Pen_Swap	2
CTents	2
motorizedbicycles	2
bassfishing	2
cosplay	2
simps	2
yugioh	2
freefolk	2
CryptoCurrencies	2
sleep	2
ar15	2
PostHardcore	2
WWEGames	2
PickAnAndroidForMe	2
DarlingInTheFranxx	2
rulesofsurvival	2
weekendgunnit	2
ps3homebrew	2

SocialEngineering	2
fo76	2
DrumkitTrading	2
AdventureCapitalist	2
weddingplanning	2
Cash4Cash	2
freestickers	2
thingsforants	2
Bape	2
gay_irl	2
SSBM	2
agt	2
csuf	2
CraftBeer	2
Dynavap	2
JustBootThings	2
Fireteams	2
girlsinyogapants	2
TeamRedditTeams	2
suicidebywords	2
vinyl	2
vlog	2
yesyesyesno	2
teefies	2
Blackbear	2
LetsNotMeet	2
kings	2
FulfillmentByAmazon	2
HaggardGarage	2
jesuschristreddit	2
adultswim	2
CoutureReps	2
h3h3_productions	2
MathHelp	2
google	2
PenmanshipPorn	2
linux4noobs	2
BBQ	2
sarmsourcetalk	2
Xiaomi	2
santaclaritadiet	2
mildyinteresting	2
shadowofmordor	2
IOTAmarkets	2
exoticspotting	2

ContagiousLaughter	2
GalaxyS9	2
lawofattraction	2
dogecoin	2
WhatsInThisThing	2
exmormon	2
Gotham	2
riverdale	2
Capitalism	2
Madden	2
curb	2
optometry	2
forza	2
chicagobulls	2
WhyWereTheyFilming	2
familyguy	2
columbia	2
safeorscamvendors	2
TheWeeknd	2
vegetarian	2
geek	2
Borderlands	2
ilikthebred	2
pouya	2
Coachella	2
slaythespire	2
cassetteculture	2
hoi4	2
Megaten	2
NoPoo	2
Bombing	2
FireEmblemHeroes	2
technicallythetruth	2
wowguilds	2
Israel	2
freelance	2
socialism	2
JUSTNOFAMILY	2
KarmaRoulette	2
ifyoulikeblank	2
Connecticut	2
MyPeopleNeedMe	2
4Runner	2
Acid	2
HPPD	2

ModernMagic	2
hutcoinsales	2
marijuanaenthusiasts	2
nexus5x	2
shittyHDR	2
AnimalTextGifs	2
McLounge	2
cyberpunkgame	2
skateboarding	2
AskTechnology	2
AndroidQuestions	2
casualChildAbuse	2
rawdenim	2
DBZDokkanBattle	2
NintendoSwitchDeals	2
hitmanimals	2
Shoes	2
teenrelationships	2
siacoin	2
grandorder	2
StickDoctor	2
malegrooming	2
classicwow	2
thenbhd	2
summonerswar	2
accidentalswastika	2
AerospaceEngineering	2
IWantToLearn	2
exxxchange	2
graffhelp	2
publix	2
aviation	2
flashlight	2
Thetruthishere	2
intrusivethoughts	2
playstation	2
VinylDeals	2
xbox	2
airsoftcirclejerk	2
xmrtrader	2
moped	2
bayarea	2
Tronix	2
GYM	2
nova	2

That70sshow	2
pcmods	2
Glitch_in_the_Matrix	2
BallPythons	2
PokemonGoNewJersey	2
birdswitharms	2
kahoot	2
RhodeIsland	2
JelBrek	2
BoxingStreams	2
UnsentLetters	2
dokha	2
bertstrips	2
GoCommitDie	2
Monero	2
oneplus	2
duolingo	2
photomarket	2
metaldetecting	2
arresteddevelopment	2
tennis	2
CreditCards	2
vagabond	2
eden	2
paydaybuilds	2
ExNoContact	2
furry_irl	2
Mabinogi	2
indiegameswap	2
playrustlfg	2
firstworldanarchists	2
flatearth	2
preppers	2
Denver	2
POLITIC	2
Vape_Sales	2
dontyouknowwhoiam	2
LILPUMP	2
Honda	2
FullScorpion	2
mallninjashit	2
twentyonepilots	2
cssbuy	2
piercing	2
MoonriseMusicFestival	2

karma	2
soundcloud	2
SmallYTChannel	2
AnimalsBeingJerks	2
MUTCoinSelling	2
NoSleepOOC	2
legostarwars	2
Physics	2
quotes	2
nothingeverhappens	2
feet	2
gambling	2
starbucks	2
getnarwhal	2
DoctorWhumour	2
trapproduction	2
SubredditSimMeta	2
gravityfalls	2
TheDepthsBelow	2
specializedtools	2
Rainmeter	2
Shemales	2
darkestdungeon	2
philosophy	2
drugtesthelp	2
FortNiteMobile	2
RepFashion	2
Competitiveoverwatch	2
OverwatchLFT	2
AnimalCrossing	2
misleadingthumbnails	2
AlienBlue	2
StarVStheForcesofEvil	2
Monitors	2
redditmobile	2
dancegavindance	2
megalinks	2
Weird	2
WorldofTanks	2
Supplements	2
videography	2
Frugal_Jerk	2
StockMarket	2
OpTicGaming	2
Kikpals	2

RBI	2
OSU	2
TownofSalemgame	2
massachusetts	2
waifuism	2
CalgaryFlames	2
TokyoGhoul	2
acne	2
CarAV	2
AmandaEliseLee	2
bonnaroo	2
modafinil	2
creepyasterisks	2
pawg	2
wifi	2
GlitchInTheMatrix	2
csshelp	2
LegitCheck	2
Kaiserreich	2
FantasyLCS	2
raining	2
ABoringDystopia	2
camping	2
caseclickers	2
Flights	2
snackexchange	2
kohi	2
woof_irl	2
brandnew	2
BobsBurgers	2
FuckMyShitUp	2
EDCexchange	2
zelda	2
law	2
cleanjokes	2
Republican	2
initiald	2
cincinnati	2
funhaus	2
SWGalaxyOfHeroes	2
DJs	2
Trufemcels	2
scambait	2
blackpeoplegifs	2
phoneverification	2

police	2
stopsmoking	2
montageparodies	2
southpark	2
gpumining	2
EnoughInternet	2
PoliticalVideo	2
spiders	2
whatstheword	2
3d6	2
CoDCompPlays	2
dishwashers	2
MaddenMobileH2H	2
catsthegame	2
Dachshund	2
dishonored	2
blop	2
Solving41818	2
finance	2
excgarated	2
APBioNBC	2
macdemarco	2
MinionHate	2
setups	2
Diepio	2
civ	2
subnautica	2
oculus	2
myfriendwantstoknow	2
theydidthemonstermath	2
EA_NHL	2
jailbreak_	2
corgi	2
dogpictures	2
whatsthatbook	2
TheFrontBottoms	2
flightsim	2
cowboys	2
edmprodcirclejerk	2
computerscience	2
AmIFreeToGo	2
yankees	2
fragranceswap	2
NetflixBestOf	2
SteamAccountsForSale	2

PokemonQuest	2
nursing	2
AccidentalComedy	2
dayzlfg	2
offwhite	2
VapeExchange	2
CloneHero	2
IdleHeroes	2
GTADupe	2
Bad_Cop_No_Donut	2
netneutrality	2
macbookpro	2
Welding	2
CorporateFacepalm	2
lossedits	2
assassinscreed	2
AmericanHorrorStory	2
progun	2
PSVR	2
FortniteLeaks	2
SpotifyPlaylists	2
californication	2
DynastyFF	2
trippy	2
curledfeetsies	2
melodichardcore	2
crypto	2
suggestmeabook	2
alexandradaddario	2
TeamFourStar	2
skimboarding	2
ebikes	2
dankchristianmemes	2
TrueSTL	2
SeaList	2
Neverbrokeabone	2
roblox	2
MCSXbox	2
LGBTeensGoneMild	2
KingdomHearts	2
FortnitePhotography	2
EvelynnMains	2
mtgtrades	2
fatlogic	2
StLouis	2

EdgyMemes	2
marvelmemes	2
TheArmory	2
LearnUselessTalents	2
fashion	2
NewYorkMets	2
N8theGr8	2
virtualreality	2
Cinema4D	2
VapeRequests	2
coaxedintoasnafu	2
sysadmin	2
PokeMoonSun	2
FinalFantasy	2
hamiltonmusical	2
sticker	2
gundeals	2
CommentKarma4Free	2
3amjokes	2
privacy	2
ARK	2
tuckedinkitties	2
XboxGameSharing	2
Charity	2
linuxmasterrace	2
insomnia	2
YouTube_startups	2
GTAGivers	2
AteTheOnion	2
ArchitecturePorn	2
furry	2
Drifting	2
blues	2
homelab	2
BillBurr	2
ANormalDayInRussia	2
vape_memes	2
GifRecipes	2
skyrimmods	2
Scrubs	2
Swimming	2
PleX	2
Planetside	2
germany	1
ImaginaryLandscapes	1

CarnivalCruiseFans	1
u_petropolis96	1
classic4chan	1
matt	1
Laptop	1
SonyVegas	1
predator	1
exredpill	1
groestlcoin	1
350z	1
Gloup	1
FlyingStudents	1
chemistryhelp	1
nfsnolimits	1
LastShadow	1
HecklerKoch	1
joyetechlogos	1
WhatSoNot	1
codeine	1
SuicideBoys	1
PhonesAreBad	1
moderatelyinteresting	1
MildlyVandalised	1
theislandsofnyne	1
bluetongueskinks	1
INJUSTICE	1
reptiles	1
u_pm_me_all_ur_pelfies	1
BlackDevinDomination	1
GarlicMarket	1
ketorecipes	1
CelebrityButts	1
Marvel_Movies	1
AZCardinals	1
flextweak	1
Hallmarks	1
a7x	1
chiari	1
testmybot	1
beetle	1
AatroxMains	1
dadhadwant	1
C25K	1
siberianhusky	1
jacksonville	1

CasualUK	1
VHS	1
sadcomics	1
milf	1
Billions	1
robbery	1
ihadastroke	1
scienceofdeduction	1
CannabisRoad	1
Gamecocks	1
u_SPBIDs	1
FacialFun	1
BackYardChickens	1
unpopular	1
BlackLivesMatter	1
nvcc	1
subbie	1
PinnaclePro	1
pond	1
yuri	1
DoomWadStation	1
Salvia	1
PERSoNA	1
Nationals	1
playpostknight	1
letscompose	1
weedstocks	1
MemoryDefrag	1
brokengifs	1
FlarrowPorn	1
curlyhair	1
stripe	1
WaxPens	1
SnapchatArt	1
Animesuggest	1
MCPE	1
Tiresaretheenemy	1
architecture	1
EmmaWatson	1
F13thegame	1
CoilGore	1
LeagueofFailures	1
Skookum	1
cad	1
nsfw_wtf	1

ced	1
solotravel	1
7daystodie	1
funrun	1
Gemini	1
KenM	1
ekkomains	1
cosplaygirls	1
felly	1
u_NebEdits	1
RBA	1
DontFundMe	1
MonsterHunterWorld	1
Epilepsy	1
InvestmentClub	1
migraine	1
theapplehub	1
hawks	1
treecaching	1
karthusmains	1
u_codeineplug	1
u_JuulFiene	1
sync_ios	1
Justridingalong	1
JediDouche	1
gorillaz	1
passionx	1
GiantCesspool	1
IDontWorkHereLady	1
OsuSkins	1
ShingekiNoKyojin	1
pewdiepie	1
ww2	1
ShrugLifeSyndicate	1
Clairvoyantreadings	1
SamONellaAcademy	1
Kava	1
angelsbaseball	1
punchableface	1
youtubesyllables	1
antivirus	1
music_like_this	1
computerhelp	1
FindTheSniper	1
vaporizer	1

showerbeer	1
japancirclejerk	1
Demonoid	1
u_Doppeltime	1
residentevil	1
Empaths	1
u_bighammer88	1
BreedingDittos	1
frogs	1
u_weihaomeva	1
riddles	1
Internet_Box	1
etherium	1
Guildwars2	1
teenmom	1
LorienLegacies	1
LittleBritt	1
unt	1
waterpolo	1
Beartooth	1
traps	1
MTB	1
net_neutrality	1
encryption	1
LiverpoolFC	1
IncelTears	1
community	1
ImaginaryColorscapes	1
BeautyBoxes	1
bbuk	1
LEDgrow	1
hansenvspredator	1
Colby	1
RateMyNudeBody	1
Wrangler	1
IKEA	1
Za_Naito	1
WTFhumor	1
SuperShibe	1
PipeTobacco	1
needadvice	1
steak	1
schizoaffective	1
trainhopping	1
Oldschool_Runescape	1

MarioTennis	1
geekbonersNSFW	1
u_Lyellae	1
forgery	1
CombatFootage	1
UnexpectedDDLC	1
IggyAzaleasAss	1
kfeets	1
MarchAgainstTrump	1
Winnipeg	1
AutoDetailing	1
blackbookgraffiti	1
tulsa	1
u_watchursix	1
mountaindew	1
htc	1
transgendercirclejerk	1
tommos	1
tails	1
Maya	1
Snubbies	1
CYBERPOWERPC	1
IsolatedVocals	1
ImHigh	1
atheistmemes	1
vndevs	1
iofferandaliexspress	1
drawing	1
typo	1
Sup	1
ShowerGoon	1
islam	1
voetbalnieuws	1
garlicstore	1
RobinhoodReferral	1
SanMarcosCA	1
churchofroyalplayer	1
CompetitiveWoW	1
BreadStapledToTrees	1
RemasterMotD	1
ypppppppppppp	1
vlone	1
GoogleAnalytics	1
RoyaleRecruit	1
LaserDisc	1

penguin	1
TheExpanse	1
alcoholism	1
rubyonrails	1
Sprint	1
noice	1
MailMonday	1
rccars	1
SNLirl	1
HelloInternet	1
fragrancecirclejerk	1
u_imguralbumbot	1
sexygirls	1
bourbon	1
HeroesandGenerals	1
HomeServer	1
animenocontext	1
Dodgers	1
JoeyDiaz	1
SneakyBackgroundFeet	1
gwent	1
EricSneakers	1
TotesMessenger	1
patientgamers	1
skycoin	1
DeliciousTraps	1
EndlessLegend	1
GTAMarket	1
santacruzlocals	1
vintagecomputing	1
jewelry	1
NFA	1
whiskey	1
ShakeAndVape	1
percussion	1
emetophobia	1
facebookwins	1
amishadowbanned	1
ents	1
askreddt	1
secretsanta	1
Glitchhop	1
NikkiBenz	1
NeutralPolitics	1
luciomains	1

vivaldibrowser	1
SteamHeads	1
ChristianGirls	1
feckingbirds	1
BeardedDragons	1
PKRepsCommunity	1
stevenuniverse	1
AskGames	1
SoccerBetting	1
bowlingforsoup	1
lggwatch	1
AskMeddit	1
DallasFuel	1
chemicalreactiongifs	1
qotsa	1
boltedontits	1
hiking	1
GamingRoomSetups	1
Animoments	1
Quinnipiac	1
kpopfap	1
MyCherryCrush	1
CaseClickerTrades	1
ReversedGIFS	1
me_ira	1
daddit	1
scholarships	1
Nimiq	1
VinylCollectors	1
relaxedpokemontrades	1
Pins4Skins	1
ThreadKillers	1
northcounty	1
u_Nilpatel7	1
DIY_eJuice_Help	1
ByteBall	1
omise_go	1
WedgieGirls	1
HaveWeMet	1
30ROCK	1
fnv	1
HongKong	1
CollegeTransfer	1
Gnostic	1
Sinemia	1

OpenEmu	1
abandoned	1
actuallesbians	1
weightroom	1
Safeway	1
shitpost	1
Nexus5	1
FRC	1
redacted	1
Fantasy	1
Habits	1
intermittentfasting	1
Roast_Me	1
mildhighclub	1
yappy	1
FriendshipAdvice	1
MLBStreams	1
france	1
StudentNurse	1
hardwaregore	1
BeatMarket	1
Psychic	1
canon	1
DesignMyRoom	1
wholesomegreentext	1
Militaryfaq	1
tressless	1
Slackline	1
fizzmains	1
tinytower	1
shockwaveporn	1
Barcelona	1
TRADE	1
forsale	1
Tennessee	1
prisonarchitect	1
pokemongotrades	1
altima	1
basketballshorts_CSS	1
BBW_Chubby	1
COMPLETEANARCHY	1
NoJumper	1
adhd_anxiety	1
HSTrack	1
noshitouija	1

Needafriend	1
AbyssRium	1
osheaga	1
toyexchange	1
AppleMusic	1
okc	1
bodyweightfitness	1
CrappyDesign2	1
Pokemongiveaway	1
Porsche	1
MilSim	1
playhitbox	1
amibeingdetained	1
computerviruses	1
VRchat	1
vermont	1
design_critiques	1
gradadmissions	1
simracing	1
sadmusic	1
raimimemes	1
Animewallpaper	1
nashville	1
poker	1
SFShock_OW	1
chronotrigger	1
pokemongobotting	1
Achievement_Hunter	1
Ballers	1
MMORPG	1
netorare	1
ps4homebrew	1
Breckenridge	1
sweepstakes	1
southafrica	1
medlabprofessionals	1
unexpectedjihad	1
Feminization	1
ColoradoRockies	1
BuzzFeedUnsolved	1
Bluzelle	1
Objectivism	1
test	1
ShittyVeganFoodPorn	1
greysanatomy	1

Michigan	1
neoncities	1
Holly_Peers	1
Naturalhair	1
Undertale	1
GamerPals	1
InterdimensionalCable	1
ShaneDawson	1
2cb	1
Testingformodstuff	1
imdbvg	1
MagicArena	1
notlikeothergirls	1
northernlion	1
reddeadredemption	1
FortniteFleaMarket	1
karmawhore	1
ramen	1
timelapse	1
fashionsouls	1
dank_memes_archive	1
needforspeed	1
darksoulspvp	1
badkarma	1
homemadexxx	1
gamecollecting	1
LogHorizon	1
MusicWorldTradeCenter	1
hmm	1
NintendoMemes	1
u_BoomXhakaLacaa	1
hyptoheicla	1
Audi	1
medicine	1
RATS	1
PumpItUp	1
JailbreakSwap	1
clusterheads	1
mattcolville	1
HeadphoneAdvice	1
FindAUnit	1
steamboat	1
TropicalWeather	1
legaladviceofftopic	1
TransyTalk	1

ufc	1
clicksforbeermoney	1
philmont	1
avb	1
VASSAL	1
abv	1
BloonsTDBattles	1
thebuttontheories	1
liltracy	1
MatthewTellis	1
moviescirclejerk	1
snowboardingnoobs	1
u_smoke_man	1
AskMenAdvice	1
PCSX2	1
AMG	1
23andme	1
OTMemes	1
USC	1
Dreadlocks	1
SkrillexDev	1
Tippmann_airsoft	1
ElectroBOOM	1
Referral	1
LRPresetSharePlace	1
ClassicalMemes	1
Cornell	1
Innokin	1
outlast	1
upvoteexeggutor	1
LodedDiper	1
Eragon	1
DIYclassifieds	1
peasants	1
ClothedTitfuck	1
filmmakinghs	1
NBA2KDesign	1
oddlyterrifying	1
NBA2kTeamUp	1
u_phasedkitten	1
techtheatre	1
humblebrag	1
OctobersVeryOwn	1
gonewildstories	1
dirtysmall	1

ledgerwallet	1
SFWporn	1
ModelUSGov	1
18_19	1
leopardgeckos	1
Substratum	1
musclecar	1
Dota2Trade	1
gamegrups	1
firstaid	1
bootlegmtg	1
javascript	1
transvoice	1
TankPorn	1
wrongnumber	1
mgmt	1
Gangstalking	1
sociopath	1
BassCanyon	1
Electrum	1
BitchImATrain	1
clevelandcavs	1
noah	1
kicksmarket	1
Shroud	1
SydneySierota	1
CopOrNot	1
RisingStorm2	1
hackernews	1
timberapp	1
Schizotypal	1
GravesMains	1
EverWing	1
inthenews	1
Overgrowth	1
Aleague	1
CroppedNorrisJokes	1
lgg6	1
Urbex	1
sleeptrain	1
98Rock	1
things	1
tomorrow	1
blender	1
spikes	1

LinusTechTips	1
INeedAName	1
telltale	1
PlayOn	1
BeamNG	1
futurebeats	1
Periods	1
policeporn	1
KatowiceTrading	1
ender3	1
india	1
PentucketHappenings	1
limerence	1
AsianCumsluts	1
LaptopDeals	1
ecr_es	1
vine	1
The_Crew	1
FUTMobile	1
ecycle	1
newburyport	1
DenverBroncos	1
Marquette	1
SDSU	1
IsThatCUM	1
FocusST	1
poshmark	1
nba_2k17	1
minnesotavikings	1
AmandaCerny	1
vitahacks	1
u_noemiecc	1
GOtrades	1
rustfactions	1
Wolfenstein	1
basketballjerseys	1
vexillology	1
simpsonswave	1
Microcenterproxy	1
woosh	1
DanmachiMemoriaFreeze	1
BoostLife	1
themastersschool	1
OCPoetry	1
boatbuilding	1

WhatsWrongWithYourCat	1
menwritingwomen	1
poop	1
lorde	1
Colorado	1
gmu	1
PaintballBST	1
Beer_Money	1
Vent	1
LOLStreams	1
EatCheapAndHealthy	1
DevilMayCry	1
policeuk	1
Forex	1
blackchickswhitedicks	1
virginvschad	1
ShittyTodayILearned	1
JustUnsubbed	1
gank	1
iOS12	1
historyteachers	1
CLOUDS	1
onceheroes	1
RandomActsOfMuffDive	1
GoogleMaps	1
SmiteLFM	1
u_NaClDaddy	1
NameMyCSGOItem	1
SurrealApprovals	1
WondersOfChina	1
whitesox	1
wowraf	1
AutoNewspaper	1
iPhone8	1
PokemonPlaza	1
DestinyGaymers	1
note8	1
GolfClash	1
shortfinal	1
CrackStatus	1
SmiteTrades	1
DatV	1
celeb_redheads	1
cryptomarket	1
DarkWebLinks	1

Domains	1
leangains	1
phillies	1
PredictMe	1
comicbookcollecting	1
fakealbumcovers	1
weirdwikihow	1
TooMeIrlForMeIrl	1
Defenders	1
powerlifting	1
Fiveheads	1
7kglobal	1
unethicallifehacks	1
ExpandDong	1
barstoolsports	1
SocialMediaIsCancer	1
Bandnames	1
Summer	1
hondaprelude	1
Dallas	1
hiphop101	1
ParaPots	1
Hype	1
SmokerHate	1
EthereumClassic	1
pokemonrmt	1
StopSpeeding	1
whothefuckup	1
Thrifty	1
Droplet_coin	1
storj	1
TEOTFW	1
Homeplate	1
usu	1
u_station_nine	1
animation	1
tvshow	1
iOSsetups	1
tucker_carlson	1
PropagandaPosters	1
dogs	1
13ReasonsWhy	1
PrintedMinis	1
pussy	1
DreamInterpretation	1

GreekMythology	1
collapse	1
Torontobluejays	1
LinkinPark	1
Vindictus	1
TestSubReddit_	1
pussypassdenied	1
GPGpractice	1
LIents	1
identifythisfont	1
adventuretime	1
MoneroMining	1
roswell	1
Lightbulb	1
amateur_boxing	1
zillakami	1
kybernetwork	1
LegitChecker	1
redditdev	1
Acceleracers	1
UnexpectedJoJo	1
petpeeve	1
S2000	1
Inventit	1
SouthLakesHS	1
Sovreignty	1
lastimages	1
Foodforthought	1
Nexomon	1
warwickmains	1
Craigslist_Cars	1
powersaves3ds	1
overcominggravity	1
cryptography	1
pencils	1
splatoon	1
jetblue	1
CricketWireless	1
funkoswap	1
syriancivilwar	1
LilYachty	1
holdmyfeedingtube	1
ecig_vendors	1
AskingAlexandria	1
uBlockOrigin	1

Portlandia	1
DetectiveConan	1
opiates_gonewild	1
pcpartpickerbuilds	1
52book	1
PVBand	1
S7Edge	1
DIYELIQUIDRECIPES	1
doggos	1
StardustCrusaders	1
cymbals	1
CrossView	1
Chevrolet	1
dailydabbers	1
FUCKDAVIDKING	1
rapbattles	1
EarthlingPorn	1
piercetheveil	1
DXMMusic	1
dubstep	1
KithNYC	1
BulkOrCut	1
problemgambling	1
NikolasCruz	1
earshit	1
StuffOnCats	1
Kendra_Sunderland	1
MUTTalk	1
sexover30	1
cinci_pokemon_go	1
thingsapp	1
BayAreaEnts	1
ChronicPain	1
LigaMX	1
Rapekink	1
StarWarsForceArena	1
xymoxcirclejerk	1
showertoughts	1
FullShrimp	1
ComputerEngineering	1
askmedicine	1
scamming	1
RedditSilverRobot	1
needamod	1
phoneswap	1

lockpicking	1
TombRaider	1
Shittyaskflying	1
Epicduck	1
Gonewild18	1
helloicon	1
Infographics	1
nyctreess	1
olemiss	1
ourgratitudejournal	1
MeetNewPeopleHere	1
PLC	1
HomeImprovement	1
coolguides	1
PartyParrot	1
Freeform	1
johannesburg	1
XVcrosstrek	1
Guilt	1
pueblo	1
gokarts	1
Megumin	1
AppleWhatShouldIBuy	1
guessmyage	1
delusionalartists	1
FitFI	1
askportland	1
dyinglight	1
SVU	1
Kanye_2	1
horror	1
ualbany	1
FIFA17UT	1
drugcirclejerk	1
ecommerce	1
PlantedTank	1
environment	1
PixelStarships	1
VideoEditing	1
newhaven	1
dogswearinghats	1
PatriotsvLionStream	1
americanfootball	1
fetish	1
twerking	1

bindingofisaacseeds	1
YungLean	1
ReelToReel	1
EngineeringPorn	1
writingprompt	1
WWE	1
Keto_Food	1
gethightothis	1
PornGifs	1
StoriesFromYourSchool	1
omegle	1
latterdaysaints	1
cosmoandwanda	1
sighthounds	1
Proxysitelists	1
TheRustyToaster	1
ArtPorn	1
kravmaga	1
vail	1
RiftForSale	1
usedsocks	1
IOTAFaucet	1
gentlefemdom	1
UpvotedBecauseMeeseek	1
PurplePillDebate	1
SparrowXXX	1
MovieSuggestions	1
MonarchButterfly	1
qdoba	1
sneakpeekbot	1
galiomains	1
logorequests	1
u_Trek1812	1
SnooRequest	1
fullmoviesongoogle	1
nationalguard	1
HearthstoneMexico	1
Lil_uzi_vert	1
wherewasthistaken	1
learnmath	1
MumkeysAnimeReviews	1
BenedictCumberbatch	1
FortniteXbox	1
sanfrancisco	1
ballpython	1

Gamecube	1
TrueFMK	1
JuulMiddleMen	1
TalesFromAdultStores	1
FancyFollicles	1
Roku	1
chrome	1
Cod4Remastered	1
Scotland	1
AnthemTheGame	1
tgn8r	1
CrackWatch	1
deathgrips	1
fappeningdiscussion	1
ToolBand	1
KLCherokee	1
FairfieldPrep	1
8tbEasystore	1
ATC	1
TampaBayLightning	1
pokemongodev	1
pocketoperators	1
OITNB	1
shia	1
vostok	1
porterrobinson	1
TheBarbarianClan	1
diyaudio	1
merchantthegame	1
KassadinMains	1
Nsfw_Haiku	1
goldenretrievers	1
HondaCB	1
ChicoCA	1
Chubbs	1
scoliosis	1
NHLStreams	1
bermuda	1
Ouija	1
Ferrari	1
PromoteYourMusic	1
KGATLW	1
TheNewRight	1
WildStar	1
MarvelStrikeForce	1

Slitherio	1
airguns	1
PurseIO	1
HunterXHunter	1
BobbyandtheBluejays	1
SecurityAnalysis	1
predictit	1
2b2t	1
GarrysMod	1
shower_thoughts	1
whatbugisthis	1
Nightmares	1
Thighs	1
BeMeApp	1
mildypenis	1
orlando	1
JETWaterPipes	1
KissAnime	1
Frisson	1
TABG	1
BenignExistence	1
pkmntcgreferences	1
reddits	1
cuddlebuddies	1
WRX	1
SUBREDDITNAME	1
shittylifehacks	1
thatsthejoke	1
ZonaEnts	1
makingmoney	1
ImagesOfMinnesota	1
Shroom	1
StudentLoans	1
microphones	1
modsarehomosexual	1
EasonyesSneakers	1
Hotchickswithtattoos	1
Wellworn	1
futurebass	1
PsilocybinMushrooms	1
chillstep	1
cripplingalcoholism	1
killthecameraman	1
photoshop	1
Vaingloryguildhall	1

vintageaudio	1
webmarketing	1
u_GalPolBlue	1
ketodrunk	1
Advance_Wars	1
nohomo	1
Shortfilms	1
ShittyCarMod	1
kotk	1
moralorel	1
cryptofit	1
HistoryPorn	1
WildernessBackpacking	1
tameimpalacirclejerk	1
rpdrcringe	1
TameImpala	1
aoe2	1
SheikMains	1
NYKnicks	1
The_Dennis	1
videogames	1
knifecringe	1
osugame	1
DuelLinks	1
6ix9ine	1
ENFP	1
thedonald	1
ResearchSourcing	1
beer	1
subway	1
PMOfreeTeens	1
LOLscrims	1
psychadelics	1
scuba	1
iPAHub	1
Vapistan	1
cloudygamer	1
redditgetsscribed	1
LeafyIsHere	1
eliaszjm	1
DeadBedrooms	1
awwcoholics	1
asl	1
infp	1
diabetes_t1	1

sharpobjects	1
Vapers_GoneWild	1
GuildWars	1
PhillyUnion	1
Homebrewing	1
FortniteAfterDark	1
EDMProductionTeam	1
crappymusic	1
UniversalOrlando	1
PSP	1
MECoOp	1
CreepyPastas	1
RetroArch	1
bikerace	1
electronic_circuits	1
badMovies	1
seniorkitties	1
Incelselfies	1
PinkOmega	1
Jetpackfighter	1
SteamBegging	1
NarcissisticAbuse	1
ParkCity	1
u_Titobanana	1
volleyball	1
tf2trade	1
totallylookslike	1
PokemonTCG	1
NZXT	1
cloudmining	1
UnreleasedSongs	1
csusm	1
lakers	1
CSUC	1
AverageBattlestations	1
Overwatch_Memes	1
cicada	1
gofundme	1
SpotifyPlaylistShare	1
StardewValley	1
breastfeeding	1
thalassaphobia	1
occult	1
APUSH	1
jimmyjoy	1

asmr	1
wineskin	1
TheEricAndreShow	1
ccna	1
CrazyHand	1
Howsmytire	1
wifepictrading	1
USF	1
taekwondo	1
phish	1
coincollecting	1
AppleBandMarket	1
AngelaWhite	1
Witcher3	1
punk	1
raspberry_pi	1
wincest	1
RandomActsOfBlowJob	1
wsb	1
autorepair	1
WrestleWithThePackage	1
StonerPhilosophy	1
Louisvuitton	1
FragDecants	1
deletefacebook	1
StopGaming	1
asianpeoplegifs	1
csgolounge	1
MCFC	1
CannabisEntrepreneurs	1
TheTwitterFeed	1
JOIP	1
paydaytheheistmods	1
bouldering	1
PublicFlashing	1
jerma985	1
NiceMods	1
Shadman	1
notebooks	1
quiver	1
NintendoSwitchDesigns	1
SubstratumNetwork	1
SNSD	1
Design	1
forhonorknights	1

49ers	1
lotr	1
SimCity	1
Nirvana	1
Titties	1
FinancialPlanning	1
Heavymind	1
florida	1
aSongOfMemesAndRage	1
supremereps	1
statenisland	1
KeepOurNetFree	1
fit	1
lululemon	1
bikewrench	1
fragsplits	1
DrugArt	1
smashgifs	1
BTD5	1
StormAge	1
ottawa	1
skytrader	1
PanoramaFest	1
cartoons	1
wedding	1
hillaryclinton	1
rickandmortytheory	1
america	1
conceptart	1
pool	1
chromeos	1
virtualdj	1
despacito	1
DumpsterSluts	1
cybermonday	1
Pokemontutoring	1
Twixtor	1
dragonquest	1
sugarfreemua	1
BitMEX	1
UnexpectedThugLife	1
ConanExiles	1
Madlib	1
Miami	1
waltonchain	1

bois	1
religion	1
musicmarketing	1
bugs	1
AerialPorn	1
hopelesssofrantic	1
tax	1
whatsthisworth	1
UCSantaBarbara	1
boogiemusic	1
pixel_phones	1
FortniteLFP	1
WizardofLegend	1
Soda	1
jimmyjohns	1
wowthanksimcured	1
lgv20	1
startrek	1
VanishingTech	1
ImagesOfNewYork	1
FifaMobileBuySell	1
BadMensAnatomy	1
lolgrindr	1
offlineTV	1
DnDGreentext	1
FestivalTrees	1
Amnesia	1
Chiraqology	1
Crouton	1
whitepeoplegifs	1
NCSU	1
ultimate	1
hentaivids	1
FootFetish	1
armpitfetish	1
Balls	1
u_b-a-b-a-y-e-e	1
u_DapDonut	1
steroids	1
costochondritis	1
pokemonduel	1
IreliaMains	1
CarAudioFabrication	1
ShittyArt	1
typewriters	1

boobbounce	1
festivals	1
ShitPoliticsSays	1
KerbalSpaceProgram	1
highseddit	1
MCPEfamily	1
normalnudes	1
Bodybuildingforum	1
Trumpgret	1
steam_giveaway	1
twitchplayspokemon	1
plexshares	1
submechanophobia	1
thebutton	1
TheForest	1
altcoin	1
Unboxtherapy	1
imsa	1
maybemaybemaybe	1
hawwkey	1
Mydaily3	1
SoftwareEngineering	1
valve	1
SugarPine7	1
pokemongola	1
BudgetAudiophile	1
ToyotaSupra	1
ULgeartrade	1
coopplay	1
TweakBounty	1
trumpet	1
KidCudi	1
BoundlessVapes	1
cade	1
send_nudes	1
wisconsin	1
u_IamdudumAusculto	1
LowTierTradingRL	1
ACPocketCamp	1
freebietalk	1
AskHealth	1
ICanDrawThat	1
pharmacy	1
DIY_eJuiceExchange	1
UpvotedBecauseButt	1

eleaf	1
ausents	1
reddit_silver	1
shortstories	1
Handwriting	1
cozypubs	1
freemoney	1
classiccars	1
ItWasMyFriend	1
AzureLane	1
Xcom	1
streetwear_memes	1
DokkanBattleCommunity	1
googlecloud	1
u_Shadow_Wolf0246	1
amihot	1
nintendolabo	1
Romania	1
angry	1
titanfolk	1
SluttyConfessions	1
futurefunkairlines	1
bloomington	1
Ravenfield	1
u_jianyang1337	1
Southerncharm	1
Mariners	1
RCycling	1
u_ogDings02	1
ProtonMail	1
lotrmemes	1
oddlyweird	1
physicaltherapy	1
MarkMyWords	1
infiniti	1
kindafunny	1
HeavySeas	1
JiggleFuck	1
backpacking	1
HealthInsurance	1
pksp	1
FlashGames	1
H1Z1OnPS4	1
ParisDylan	1
AskVet	1

anime_irl	1
DrugNerds	1
StraightGirlsPlaying	1
ohwonder	1
NSFW_HORNY_GIRLS	1
binance	1
StreetwearLegitChecks	1
feedthebeastservers	1
tevotarantula	1
Genuine_Memes	1
BossFights	1
redditsync	1
bees	1
Sneaking	1
appdev	1
cokebears	1
fordfusion	1
SoylentMarket	1
howitsmade	1
Mixcraft_Studio	1
rockhounds	1
pixallio	1
Faces	1
ImaginaryHybrids	1
EndlessSpace	1
cannabiscultivation	1
ontario	1
Archery	1
nugporn	1
ATT	1
Allotment	1
ifiwonthelottery	1
SisyphusIndustries	1
ProperAnimalNames	1
adidas	1
wrestling	1
csgogambling	1
Invisalign	1
lol	1
unexpectedclue	1
ios7	1
CodinGeek	1
u_IamNickJones	1
ICoveredASong	1
justiceleague	1

Archeology	1
Electoral_College	1
COents	1
indianews	1
footbaww	1
Wizard101	1
Jcole	1
santacruzents	1
4x4	1
scooter	1
sportsjerseys	1
simbot	1
atheistparents	1
EllieMain	1
KnightsOfPineapple	1
IsTodayFridayThe13th	1
WatchRedditDie	1
DiagnoseMe	1
ketogains	1
knifeclub	1
NetSpend	1
shittydarksouls	1
OnBenchNow	1
scifi	1
triangle	1
Vanic	1
dashcamgifs	1
victoriajustice	1
Destiny_2	1
AmazonMerch	1
UnexpectedPrequelMeme	1
gloving	1
TeamSESH	1
u_MickeyReddit2016	1
ShittyPickupLines	1
Toonami	1
GroupOfNudeGirls	1
RutgersNewark	1
dnp	1
Traderfeedback	1
AbsoluteUnits	1
NBAForums	1
TarkovTrading	1
PRXJEK	1
emulation	1

beards	1
heat	1
AsianAndy	1
sffpc	1
firstimpression	1
shrug	1
Nioh	1
CastleClash	1
redditrequest	1
e30classifieds	1
bee_irl	1
Momokun_MariahMallad	1
HomeDepot	1
amipregnant	1
CanadianMOMs	1
fairytail	1
thebachelor	1
Lexus	1
morebreedingdittos	1
villanova	1
LV426	1
homework_help	1
WisconsinBadgers	1
TalkWithSomeone	1
badbadnotgood	1
FierceFlow	1
foshelter	1
reverselegaladvice	1
Bioshock	1
CompanyBattles	1
rwallpaperchanger	1
RandomActsOfPolish	1
CartoonNetwork	1
CaseClickerChannels	1
CaliPedes	1
NoContract	1
WeightTraining	1
trackers	1
questions	1
urbantees	1
soulseeker	1
VPS	1
EnterTheGungeon	1
SMU	1
HansaDarknetMarket	1

u_affcashmania	1
geography	1
led_zeppelin	1
LSDGamers	1
DoMyHomework	1
gunship	1
cbdjuul	1
shiba	1
DCents	1
mountandblade	1
comedyhomicide	1
BokuNoMetaAcademia	1
PiratedGTA	1
CZFirearms	1
berlin	1
Catholicism	1
grammar	1
PuffinbirdAlogRS	1
GEazy	1
cute	1
curvy	1
DeathGripsCirclejerk	1
androidroot	1
mylittlepony	1
seemslegit	1
boardgames	1
WTFgaragesale	1
CelebrityFeet	1
NSFW_GIF	1
backgammon	1
Mercerinfo	1
scfeedback	1
FalloutMods	1
BringMeTheHorizon	1
handguns	1
coupons	1
Negareddit	1
Truckers	1
gayyoungold	1
Clerks	1
BitcoinPrivate	1
whatsthisfish	1
shroomers	1
IdiotsFightingThings	1
PokeGo2	1

KiraKosarin	1
electriczoo	1
KarmaStore	1
ukulele	1
battleshops	1
dtvn_accountmarket	1
hughmungus	1
OverwatchTMZ	1
behindthegifs	1
Homebrews	1
leakthreads	1
RocketLeaguePS4	1
VertcoinMining	1
Drugtest	1
AskAnAmerican	1
Project_Badass	1
Pathfinder_RPG	1
screenshots	1
twitchstreams	1
Miscreated	1
cookiedecorating	1
vandwellers	1
Twokinds	1
DrunkText	1
ChrisChanSonichu	1
algotrading	1
SFGSocial	1
doodles	1
Sannatorr	1
Timberland	1
Metallica	1
Nexus6P	1
coys	1
nbastreams	1
Borderlands_3	1
Inventions	1
minishibe	1
Parkour	1
90sdesign	1
Hmmmmm	1
meme_hell	1
BoardGameExchange	1
april30th2015	1
penguins	1
Anticonsumption	1

u_Seemslikesam	1
classicalmusic	1
hisnameishb	1
mercedes	1
FiestaST	1
PAWGtastic	1
u_khuber123	1
Emuwarflashbacks	1
bigtiddygothgf	1
altrap	1
rcdrift	1
RocketLeagueSexChange	1
u_tallglassofH2O	1
scenes	1
WorldOfWarships	1
Pontiac	1
bingingwithbabish	1
cults	1
WoahTube	1
luigi_irl	1
TinyGladiators	1
minimalism	1
JhinMains	1
panoramicsgonewrong	1
hiphopvinyl	1
crusadersquest	1
LipsThatGrip	1
womensstreetwear	1
StonerThoughts	1
CRH	1
SarahRoseMcDaniel	1
csgocritic	1
fragrance	1
DDLCMods	1
chillout	1
firstworldproblems	1
singapore	1
CrackheadCraigslist	1
UBreddit	1
ThisIsOurMusic	1
PoliceVehicles	1
learningcss123	1
KnifeRaffle	1
u_RavageBladez	1
FalcoMains	1

DeformationHentai	1
marthasvineyard	1
ptcgo	1
firefighters	1
0xbitcoin	1
Pay_Respects	1
Favor	1
CompetitiveMinecraft	1
tripreports	1
youseeingthisshit	1
prisonhooch	1
Volkswagen	1
medievaldoctor	1
breathinginformation	1
swedishsnus	1
SteamMarket	1
iwatchedanoldmovie	1
DestinyDadJokes	1
fraud	1
Pyongang	1
manchester	1
PurpleCoco	1
Career_Advice	1
Artifact	1
camarillo	1
Howwastoday	1
3dspiracy	1
Smartphones	1
ETNmining	1
Adoption	1
u_MexkeyJuan	1
KanyeLeaks	1
SchwiftyIDs	1
awardtravel	1
BoarVesselMemes	1
GoneMild	1
Anarchism	1
WeedWiki	1
FuckYou	1
wesanderson	1
Preparedness	1
SCU	1
picrequests	1
AtheisticTeens	1
girlsfarting	1

czscorpion	1
H1Z1LFG	1
21savage	1
Nexus	1
FreezingColdTakes	1
HadToHurt	1
SteamTradingCards	1
football	1
heep	1
Lightning	1
AtlantaHawks	1
fortniteSSD	1
CryptoWorth	1
Perfume	1
mcpublic	1
jtag360	1
getkarma	1
u_rosssands4	1
huffing	1
noxappplayer	1
tailosive	1
redneckengineering	1
Network	1
RealAhegao	1
whitepeoplefacebook	1
tattoo	1
Hunting	1
gamingsetup	1
scrapinghub	1
pools	1
u_pawgnation462	1
sousvide	1
flavortown	1
goodkarma	1
cumsluts	1
ClassicNation	1
nedforpresident	1
Voting	1
aphextwin	1
mazda	1
catsareliquid	1
notdisneyvacation	1
ExpanseOfficial	1
FeetToesAndSocks	1
snapsext	1

Hawaii	1
AskManagement	1
Chattanooga	1
fleshlight	1
VirtualCosplay	1
nsfw_video	1
crappycontouring	1
bingbongtheorem	1
PoliticalScience	1
lifeisstrange	1
Viceland	1
csharp	1
Amtrak	1
NormMacdonald	1
Internet_Strangers	1
FuckTanya	1
9gag	1
czech	1
CountOnceADay	1
gangplankmains	1
fantasybaseball	1
Batch	1
Stock_Picks	1
BubbleHash	1
CherokeeXJ	1
gamingcomputer	1
3atatime	1
Scary	1
pihole	1
flightattendants	1
RedditLaqueristas	1
mcgill	1
controllablewebcams	1
Greek_Mythology	1
Overwatch_Porn	1
oklahoma	1
Dudeism	1
XboxModding	1
Awww	1
battlefield3	1
HealthAnxiety	1
redditlogos	1
PartneredYoutube	1
Stellar	1
budgetdecks	1

hometheater	1
Dollywinks	1
maryland	1
pokemonteams	1
WhatsThisShoe	1
Agorism	1
PC_Builders	1
JojosBizarreMemePage	1
LetItDie	1
labrador	1
ShitPostCrusaders	1
StartledCats	1
UHRSwork	1
NewGirl	1
amrc	1
ApocalypseRising	1
witcher	1
tacticalgear	1
berkeley	1
trypophobia	1
CSGOScamList	1
BitcoinMarkets	1
darknet	1
Blogging	1
troubledteens	1
BleachBraveSouls	1
PSHFlatEarth	1
latin	1
shittyideas	1
AppNana	1
Allsvenskan	1
CabaloftheBuildsmiths	1
Battlegrounds	1
IAmAFiction	1
Pets	1
sales	1
Crewniverse	1
Transmogrification	1
writingdaily	1
youtubecomments	1
PinkFloydCircleJerk	1
Culvers	1
cheesecake	1
icehockey	1
Colts	1

retrogameswap	1
Ghosts	1
deadcells	1
VolvoRWD	1
warframeclanrecruit	1
fullscorpian	1
kurdistan	1
sevenknightsfriends	1
TrollXChromosomes	1
upvote_for_upvote	1
BossfightUniverse	1
Greyhounds	1
hcfactions	1
Economics	1
nsfw	1
MeepoBoards	1
NotTimAndEric	1
relationships_advice	1
editing	1
aimdownsights	1
Unextexted	1
linuxmemes	1
DadReflexes	1
ftm	1
wedidit	1
IAmARequests	1
Anxietyhelp	1
KingOfTheHill	1
Naturewasmetal	1
LakeLaogai	1
MapleStory2	1
badselfeater	1
shittylimos	1
pokies	1
Reddit_bronze	1
CHART_BOT	1
driving	1
DexterKicksbar	1
SonyXperia	1
wallpaper	1
LegendsOfTomorrow	1
RateMeUnder18	1
pickuplines	1
Patches	1
bioniclelego	1

cycling	1
anonymous	1
graphicscard	1
NewToReddit	1
CSGOKnives	1
meth	1
ThatPeelingFeeling	1
VPNTorrents	1
stopdrinking	1
musical_instruments	1
TreesSuckingOnThings	1
bigboye	1
collegesluts	1
deals	1
NEU	1
Konosuba	1
Turkey	1
MapleStoryM	1
Austin	1
reddit.com	1
ToCatchAPredator	1
Wiffleball	1
excel	1
AMCsAList	1
Goldfish	1
beats	1
AlwaysSunnyGifs	1
SubForSub	1
mturk	1
Dankquan	1
proxies	1
riddim	1
spongebob	1
NFL_Draft	1
DesirePath	1
cannabis	1
Edits	1
LawSchool	1
anal_gifs	1
GuessTheMovie	1
Hempire	1
chemhelp	1
BeautyGuruChatter	1
adwords	1
brokenbones	1

karma4karma	1
Mauser	1
ZClassic	1
AskMechanics	1
Superbuy	1
AP_Physics	1
AsianHotties	1
nutella	1
DaveRamsey	1
Trucks	1
u_Hawk7117	1
OverwatchCompetitive	1
introvert	1
UBC	1
JacksFilms	1
predaddit	1
HSPulls	1
TopGear	1
elderscrollsonline	1
dgu	1
DarkSouls2	1
gamingsuggestions	1
demifiendnocturne	1
ubereatspromocodes	1
ModernTwin	1
GetStudying	1
electricdaisycarnival	1
Competetive_Overwatch	1
egg_irl	1
cynicalbritofficial	1
GetOffMyChest	1
Xbox_One_X	1
unturned	1
BDSMAdvice	1
ParanormalEncounters	1
Prematurecelebration	1
deadpool	1
physicsgifs	1
banme	1
MonsterHunter	1
malta	1
tacobell	1
Infinitewarfare	1
iOSProgramming	1
portabledabs	1

CompetitiveHS	1
Wawa	1
OculusStore	1
Persona4Chie	1
spicy	1
upvote	1
CalamariRaceTeam	1
u_irishAAron5963	1
EANHLfranchise	1
coincidence	1
forhire	1
Crush	1
scufgaming	1
gamingsetups	1
ImagesOfIllinois	1
whoooosh	1
SeattleWA	1
dataisugly	1
maker	1
911FOX	1
ShitMomGroupsSay	1
Gundam	1
MURICA	1
cottontails	1
yardsale	1
beholdthemasterrace	1
Sense8	1
SoundCloudHipHop	1
JeepWrangler	1
transpassing	1
FirePorn	1
JaneTheVirginCW	1
Multicopter	1
lgwatchsport	1
u_Dogacado	1
YoungThug	1
CompetitiveForHonor	1
u_GoD_pewpew	1
GetMoreViewsYT	1
intothetunnel	1
CodingHelp	1
BigBrother	1
scouting	1
The_Purple_Order	1
FortNiteLFG	1

SourceFed	1
WOWGO	1
famousdex	1
trippieredd	1
longboardingGEAR	1
FinalMouse	1
kitty	1
paxful	1
drawme	1
nxyz	1
uvtrade	1
pulpfiction	1
Golf_R	1
Reddit_Inquisition	1
KansasCityChiefs	1
MtF	1
erau	1
unexpectedpedo	1
communism101	1
immigration	1
nukedmemes	1
ExpectationVsReality	1
MASFandom	1
characterdrawing	1
DHgate	1
AFL	1
AMDHelp	1
InteriorDesign	1
burlington	1
djiphantom	1
FACEITcom	1
xqcow	1
worldbuilding	1
Phobia	1
greenday	1
YouShouldKnow	1
msu	1
quittingkratom	1
smallbusiness	1
insults	1
u_GodseySupply	1
naturalbodybuilding	1
stratisplatform	1
MyrtleBeach	1
RequestABot	1

FuckJeffSessions	1
Totallyaccuratebattle	1
EnglishLearning	1
PeakyBlinders	1
wowservers	1
Dirtykikpals2	1
sticknpokes	1
AmateurPorn	1
AlbumArtPorn	1
boburnham	1
mailroom	1
Random_Acts_Of_Amazon	1
u_the_jacob_perez	1
redheads	1
ps3hacks	1
MorbidReality	1
teen_fashion_advice	1
spiral_knights	1
Clash_Royal	1
ctbeer	1
unrealtournament	1
rheumatoid	1
eFreebies	1
amazonecho	1
darksouls	1
future_fight	1
UnnecessaryQuotes	1
Flightkickz	1
promos	1
Bellingham	1
TankiOnline	1
horizon	1
metals	1
vegas	1
OddlyArousing	1
DMAcademy	1
euthanasia	1
doctors	1
bdsmSFW	1
VACsucks	1
OneNote	1
ChivalryGame	1
hookup	1
HPHogwartsMystery	1
f150	1

LitecoinCashMarkets	1
gtafriends	1
ProshotMusicals	1
IsaiahRashad	1
RateMyGf	1
u_TrippyHippyCrew	1
Tianeptine	1
JeepLiberty	1
liluglymane	1
wikipedia	1
OhioStateFootball	1
Mcat	1
weeabootales	1
tech	1
askphilosophy	1
NSFW_GIF	1
4.82E+09	1
AtlantaTV	1
TWDRoadToSurvival	1
WGU	1
Soulnexus	1
aznidentity	1
hmmmgifs	1
shittytumblrgifs	1
AnimalsBeingDerps	1
Addons4Kodi	1
RowanUniversity	1
swoleacceptance	1
Nepal	1
LunaDragon	1
Dominos	1
lesbians	1
cocktails	1
Proofreading	1
lepin	1
Snek	1
IDmydog	1
asianpornstars	1
gonewildaudio	1
iOSHoudini	1
skateboardcirclejerk	1
allentown	1
IsTodayOppositeDay	1
legotrade	1
casualnintendo	1

GSkill	1
PokemonROMhacks	1
E30	1
HMWH	1
SexyFrex	1
TinyTits	1
Rowing	1
democrats	1
nspire	1
geekboners	1
lesbianr4r	1
TVDetails	1
Pitt	1
stickers	1
MakingIt_Show	1
waluigidid911	1
fixedgear	1
ibs	1
Birkenstocks	1
ScarySigns	1
titlegore	1
UMF	1
AskMarketing	1
education	1
GearTrade	1
Miniworlds	1
CopyPastas	1
ShortFilm	1
DannyBrown	1
whenitgoesin	1
birthday	1
Georgia	1
CollegeatBrockport	1
macgaming	1
PicsofCouples	1
RemixOS	1
serialkillers	1
actuary	1
functionaldrugs	1
Ask_Lawyers	1
GHOSTEMANE	1
Falconry	1
genderfluid	1
DebateCommunism	1
texts	1

Carving	1
counterstrikego	1
AskNetsec	1
OCLions	1
ReadyOrNotGame	1
interstellar	1
Texans	1
PokemonInsurgence	1
carscirclejerk	1
BitcoinMarkets_test	1
flyfishing	1
Toreba	1
JapanTravel	1
nameaserver	1
DirtyJokes	1
GoNets	1
SOPA	1
MegaMoney	1
PiratedGames	1
JessicaNigri	1
osureport	1
stunfisk	1
FoundPaper	1
bladeandsoul	1
DeathStranding	1
lolcats	1
gaybros	1
OriannaMains	1
CryptoTrade	1
Finobe	1
cinematography	1
love	1
CubicleWarfare	1
mechanical_keyboard	1
marchingband	1
nhl	1
cm3d2	1
sooners	1
findaleague	1
Drumming	1
Gta5Modding	1
Mushrooms	1
yiff	1
RealTesla	1
UPS	1

HollowKnight	1
AmpliFi	1
Puzzleboys	1
springfieldMO	1
newsubreddits	1
RedLetterMedia	1
G37	1
ForbiddenCandy	1
CouncilOfRicks	1
AskDoctorSmeeee	1
Ethnomethodology	1
chemistrymemes	1
JerseysClub	1
TrueDetective	1
u_BooyahAdvertising	1
kahootraiding	1
badwomensanatomy	1
Getter	1
AccidentalRenaissance	1
orioles	1
averii	1
e46	1
ROTC	1
CaptainTsubasaDT	1
SS13	1
Nicegirls	1
FifaMobile	1
Chilledout	1
bengals	1
IncestComics	1
alisio	1
TwinCities	1
philadelphia	1
UCSC	1
AmISexy	1
cscareerquestions	1
Staples	1
RetroPie	1
UFOs	1
Coilporn	1
AganistGayMarriage	1
polls	1
elonmusk	1
TruthOrTrump	1
angst	1

rocket_league_trading	1
Troy	1
BigTimeRush	1
HighQualityReloads	1
drunkandsad	1
django	1
SequelMemes	1
animegifs	1
ExplainItLikeImMorty	1
gameshow	1
xcodeh	1
User_Simulator	1
60fpsporn	1
drumcorps	1
PlayJustSurvive	1
bch	1
mehfaketexts	1
PucaTrade	1
nosneeze	1
sixthworldproblems	1
AskLE	1
COD_LFG	1
demonssouls	1
gifsthatendtoosoon	1
Irony	1
DoesSheLikeMe	1
McDonalds	1
mariorule34	1
ImagesOfOregon	1
paszagonewild	1
AskAMechanic	1
ProductPorn	1
RX7	1
mintmobile	1
supportlol	1
WendellGoldwater	1
Oswego	1
911truth	1
Iota	1
ucla	1
yescompanionimbecil	1
mashups	1
kustom	1
LeBlancMains	1
avengedsevenfold	1

playlists	1
SFGiants	1
PowerTV	1
free_karma	1
LDESurvival	1
TheLastJedi	1
keming	1
mechanics	1
HutCoinSelling	1
csgomarketforum	1
u_fireice2929	1
soccerstreams	1
factorio	1
LilSkies	1
Adirondacks	1
fights	1
hackersec	1
survivorbeauty	1
justTurnedEighteen	1
xenia	1
Remington	1
Polytopia	1
BytecoinBCN	1
USNEWS	1
yorickmains	1
NuFunk	1
Battlefield2	1
Battleborn	1
YIMO	1
bonehurtingair	1
SanJose	1
BostonU	1
azirmains	1
gopro	1
mentalhealth	1
IAmSelling	1
ipad	1
italy	1
FightH1B	1
swingersr4r	1
shittyadvice	1
CageTheElephant	1
calmhands	1
pastlives	1
GameShare	1

singing	1
PUBGXBOX	1
UCONN	1
Ultraboost	1
Steam_Link	1
oddlyunsatisfying	1
ImagesOfFlorida	1
TheVGang	1
Duolingoclubs	1
shitty_ecr	1
covers	1
weightlifting	1
MassiveCock	1
newtothenavy	1
WoT	1
dogeducation	1
GetMotivatedBuddies	1
guncontrol	1
u_Steel_Punch2	1
DeTrashed	1
edencirclejerk	1
yesthereis	1
SkiRacing	1
sunypoly	1
j_chetta	1
SissTest	1
subredditideas	1
LiveFromNewYork	1
2mad4earth	1
pancreatitis	1
UnionHouse	1
Diablo	1
IncestPorn	1
chocolate	1
HomePod	1
vapormax	1
tinnitus	1
mrpickles	1
neoliberal	1
RocketLeaguePS4Trades	1
Braves	1
blackdesertonline	1
PhantomForSnapchat	1
LisaAnn	1
WatchExchangeFeedback	1

WiiUHacks	1
entrepeneur	1
paragon	1
4hourbodyslowcarb	1
DemiRoseMawby	1
TonightShowFallon	1
Peptides	1
Snus	1
TeenMomOGandTeenMom2	1
dirtypenpals	1
Simulated	1
StupidCarQuestions	1
likeus	1
bettereveryrepost	1
playmavericks	1
akalimains	1
PrivateInternetAccess	1
UniClassof2023	1
handslikehouses	1
___	1
michaelcollins	1
sodadungeon	1
MUWs	1
SupremeResell	1
Chiropractic	1
AmericasArmyPS4	1
oracle	1
TrumpCriticizesTrump	1
spotted	1
LandCruisers	1
rearpussy	1
onewordeach	1
iPhoneography	1
PokePorn	1
eldertrees	1
riotmc	1
AskEngineers	1
AntiFlag	1
miamioh	1
PoliceChases	1
uscg	1
Nudah	1
mtgfinance	1
WoahTunes	1
fortwayne	1

Persona5	1
painting	1
trump	1
funkopop	1
PEDs	1
ButtSharpies	1
Bostontreesforall	1
seventhworldproblems	1
13or30	1
Survival	1
utdallas	1
roastmytrack	1
LucioRollouts	1
cyclocross	1
prochoice	1
paydaytheheist	1
religiousfruitcake	1
KneeInjuries	1
simpleliving	1
aberothtrading	1
survivetheculling	1
girlsdoporn	1
askdrugs	1
BuffHydra	1
ghettofunk	1
SourceLiveAudioMixer	1
ReefTank	1
TedDanzig	1
Emo	1
PHP	1
IncrediblesMemes	1
Bugatti	1
traderjoes	1
Tesla	1
gamemaker	1
iran	1
Titty_fuck_gif	1
Metal	1
DescentIntoTyranny	1
psilocybin	1
FaZe	1
startups	1
AsianMasculinity	1
UnexplainedPhotos	1
ColumbiYEAH	1

poppunkers	1
TimAndEric	1
comicbooks	1
CHIBears	1
thesopranos	1
sidetrackedday	1
iPhoneXS	1
homelabsales	1
ems	1
Nr2003	1
ProjectOblio	1
coil_building	1
curlygirl	1
bartenders	1
China	1
bepis	1
Helicopters	1
Catloaf	1
Verticalwallpapers	1
Lolice	1
BDSMpersonals	1
Antiques	1
potcoin	1
Huawei	1
removalbot	1
work	1
geometrydash	1
FCCincinnati	1
Drumkits	1
altcoinmining	1
dankruto	1
DragonballLegends	1
CanadianForces	1
arkhamknight	1
HawaiiVisitors	1
Pizza	1
aesthetic	1
ericandre	1
fakeidsuperlist	1
Harley	1
watch_dogs	1
PopCornTime	1
FantasyPL	1
CuttingWeight	1
Charcuterie	1

clevercomebacks	1
Methadone	1
Hair	1
mildlyvagina	1
cummingonfigurines	1
POTUS	1
Whistler	1
C_S_T	1
mercedes_benz	1
KatelynNacon	1
illusionporn	1
Woodcarving	1
Crunchyroll	1
MysteryDungeon	1
tesdcares	1
freaksandgeeks	1
brushybrushy	1
Cumtown	1
wiiu	1
TomatoFTW	1
mycology	1
AmsterdamEnts	1
theflash	1
XRayPorn	1
G35	1
Atkins	1
AVexchange	1
battlefield_live	1
kendo	1
japanesestreetwear	1
GitBug	1
plebflair	1
ffxivcrafting	1
Cisco	1
standupshots	1
pchelp	1
GastricBypass	1
NonZeroDay	1
spaz	1
ImaginaryWastelands	1
TheDarkTower	1
noisygifs	1
PhysicsStudents	1
Nissan	1
VapingInPhoenix	1

economy	1
perfectlycutscreams	1
1200isjerky	1
roadtrip	1
PaladinsStrike	1
edubble	1
Tarantino	1
Harvard	1
TAPBASEBALL	1
justlegbeardthings	1
NCAAFBseries	1
RightwingLGBT	1
poketcgonlinetrading	1
chillmusic	1
howtoaddict	1
Birmingham	1
battleroyalegames	1
CircLoLjerk	1
hockeygoalies	1
RobinHoodPennyStocks	1
AskSF	1
wakieshitposts	1
jetski	1
lyftdrivers	1
vapetricks	1
begleri	1
ArianaGrande	1
DnD	1
Shoplyfter	1
HairDye	1
Haircare	1
mantids	1
homemaking	1
kahootcrashing4kids	1
Attila	1
Ubuntu	1
lincolndouglas	1
diabetes	1
birthcontrol	1
lacroix	1
singlespeed	1
gaypornhunters	1
moths	1
touhou	1
StudioOne	1

Hulu		1
ClickerHeroes		1
humblebundles		1
restofthefuckingowl		1
DemiLovato		1
Craps		1
isometric		1
fivenightsatfreddys		1
frugalstreetwear		1
Atlanta		1
AirBnB		1
R6ProLeague		1
Syracuse		1
ltc		1
	1022	1
KetoAndPoor		1
dirtykikpals		1
InstagramMarketing		1
ShotGlassBets		1
TopMindsOfReddit		1
Keep_Track		1
mdlds		1
amateurradio		1
richiesnose		1
TheFacebookDelusion		1
HIMYM		1
glasses		1
snapchatgeofilters		1
wtfstockphotos		1
jacking		1
YanetGarcia		1
DaftPunk		1
asoiaf		1
UnexpectedMulaney		1
dirtgame		1
RealEstate		1
trackandfield		1
ASAPmob		1
jrotc		1
AmateurRoomPorn		1
GFRIEND		1
dating		1
YouTubersGoneWild		1
Afrodisiac		1
DCTelevision		1

SkyrimMemes	1
lightshope	1
JohnMayer	1
pottoblame	1
chachigonzales	1
MrRobot	1
theroom	1
onejob	1
Ethorse	1
Warthunder	1
JAILBREAKworld	1
ClashDecks	1
distension	1
nocopyrightsounds	1
mythology	1
mazda3	1
SoccerNoobs	1
KhaZixMains	1
KKKMOONMAN	1
QMEE	1
Thundercat	1
FFXV	1
u_shreksasscheek	1
XCOM2	1
options	1
collegeresults	1
CollegeEssayReview	1
KnifeDeals	1
2cx	1
todayiwaslucky	1
fantanoforever	1
livesound	1
SwitchFCSwap	1
fashioneconomy	1
freebeats	1
homeautomation	1
YEEZYBOOST350	1
Magicdeckbuilding	1
ToiletThoughts	1
DebateAltRight	1
Kings_Raid	1
stolendogbeds	1
PanPorn	1
Costco	1
Ipsy	1

FreeEBOOKS	1
girlsinleggings	1
ainbow	1
ejuice	1
MousepadReview	1
Norway	1
TheDarwinProject	1
Crypto_Currency_News	1
radiohead	1
recipes	1
flashcarts	1
Mammoth	1
BanBanouts	1
u_Wolfs_Taco	1
sweatcoin	1
Slayer	1
Rhinoplasty	1
Vyvanse	1
GoProMarket	1
AutoVloggers	1
EmpireDidNothingWrong	1
RedditSnow	1
TheWalkingDeadGame	1
reclassified	1
Cocofleshlights	1
grateful_dead	1
SummonSign	1
NotKenM	1
ComplimentMePlease	1
outside	1
ArtisanMacro	1
goodideas	1
dune	1
TheresARedditForThat	1
penis	1
shield	1
sailing	1
myog	1
WowUI	1
GFTJCreampie	1
conspiracytheories	1
Pauper	1
NYGiants	1
prettylights	1
u_CarlosTheCar	1

Contest	1
Fallout4Builds	1
dungeonrealms	1
LGG3	1
u_jonwong966	1
JerkOffToCelebs	1
Dx2SMTLiberation	1
olkb	1
obgyn	1
RepTronics	1
100DaysofKeto	1
BadMUAs	1
medizzy	1
glowingeyesshit	1
StoneSour	1
SmiteTactics	1
tacos	1
Health	1
OWConsole	1
FreeGamesOnSteam	1
pceo	1
dogswithjobs	1
arcticmonkeys	1
subwoofer	1
suits	1
ps4friends	1
NYCbike	1
HitBoxPorn	1
JRPG	1
CrownVictoria	1
PS3	1
Ducati	1
gtamods	1
Spanish	1
ShadowgunLegends	1
vancouverbuysell	1
LogitechG	1
TF2WeaponIdeas	1
namenerds	1
jobcorps	1
MindHunter	1
MaddieReed	1
Composing	1
racing	1
asktransgender	1

UofO	1
fishtank	1
RightRecipes	1
yunggravy	1
SonyAlpha	1
blackfriday	1
HaileeSteinfeld	1
smoking	1
premiere	1
boobs	1
Pieces	1
Electroneum	1
CiciKicks	1
duelyst	1
OverwatchLFG	1
neature	1
Firearms	1
TechNope	1
backwoods	1
Weldingporn	1
prolife	1
feetvids	1
MusicalTheatreScores	1
rock	1
Buttcoin	1
boulder	1
LateNightTalkShows	1
RealLifeLore	1
FGOcomics	1
Lehigh	1
njpw	1
catpictures	1
depression_help	1
crochet	1
biology	1
pregnant	1
GodEater	1
