# Supplementary material for: Underage JUUL Use Patterns: Content Analysis of Reddit Messages
Source: J Med Internet Res. 2019 Sep 9;21(9):e13038. doi: 10.2196/13038 (PMC6786857; doi:10.2196/13038)
Supplement: Multimedia Appendix 2 [file jmir_v21i9e13038_app2.pdf]

Appendix 2. Location summary to each state

Tennessee	3
Arkansas	0
Maine	0
Maryland	0
Oklahoma	0
Ohio	1
Arizona	2
Rhode Island	0
Colorado	4
Alabama	0
Pennsylvania	3
West Virginia	0
Illinois	1
Minnesota	1
North Carolina	1
Washington	1
New Jersey	3
Montana	0
Wisconsin	1
North Dakota	0
Hawaii	1
Nebraska	0
Kentucky	0
New York	11
Louisiana	0
Texas	6
Idaho	0
Nevada	0
Oregon	1
Vermont	0
Alaska	0
Florida	5
Michigan	2
Virginia	1
Georgia	1
Utah	1
Delaware	0
South Dakota	0
Kansas	0
Missouri	3
New Mexico	1
Connecticut	1
Iowa	0

Indiana	0
New Hampshire	0
South Carolina	0
Wyoming	0
Mississippi	0
Massachusetts	2
California	17
